# A Systematic Review and Meta‐Analysis of Semaglutide Effects on Adipose Tissue and Emerging Effects on Brain and Cognition

**DOI:** 10.1111/obr.70108

**Published:** 2026-03-01

**Authors:** Amene Saghazadeh, Mahsa Dolatshahi, Soheil Mohammadi, Sara Hosseinzadeh Kassani, Mahshid Naghashzadeh, Joseph E. Ippolito, Claude B. Sirlin, Bettina Mittendorfer, Matthew R. Brier, Suzanne E. Schindler, John C. Morris, Danny Mou, Hani Charles Soudah, Tammie L. S. Benzinger, Cyrus A. Raji

**Affiliations:** ^1^ School of Medicine Tehran University of Medical Sciences Tehran Iran; ^2^ Mallinckrodt Institute of Radiology Washington University in St. Louis St. Louis Missouri USA; ^3^ Department of Biochemistry and Molecular Biophysics Washington University School of Medicine St. Louis Missouri USA; ^4^ Liver Imaging Group, Department of Radiology University of California San Diego California USA; ^5^ Department of Medicine and Nutrition & Exercise Physiology University of Missouri School of Medicine Columbia Missouri USA; ^6^ Department of Neurology, Washington University School of Medicine St. Louis Missouri USA; ^7^ Charles F. and Joanne Knight Alzheimer Disease Research Center (Knight ADRC) Washington University School of Medicine St. Louis Missouri USA; ^8^ Department of Surgery Washington University School of Medicine St. Louis Missouri USA; ^9^ Department of Medicine Washington University School of Medicine St. Louis Missouri USA; ^10^ Department of Neurosurgery Washington University School of Medicine St. Louis Missouri USA

**Keywords:** adiposity, BMI, dementia, diabetes, incretin mimetic, treatment duration, VAT area, visceral obesity

## Abstract

**Background:**

In 2023 and 2024, research into semaglutide (SEMA), an antiobesity and antidiabetic medication, indicated potential benefits beyond its approved uses, particularly in preventing Alzheimer's disease (AD). This highlights the link between obesity and AD development.

**Objectives:**

This systematic review and meta‐analysis evaluates clinical studies assessing SEMA's effects on subcutaneous and visceral adipose tissue (SAT/VAT) measures, brain function, cognitive performance through cognitive tasks, and the incidence of cognitive disorders.

**Methods:**

We searched databases for studies evaluating these outcomes pre‐ and post‐SEMA treatment, with the last update on November 9, 2024. We included studies regardless of treatment duration, estimating pre‐post standardized mean differences (SMD) for one‐group and two‐group designs using a random‐effects model.

**Results:**

We included 23 studies: 18 on SAT/VAT outcomes and five on brain function and cognitive impairment. Meta‐analyses revealed significant VAT reductions but no significant impact on SAT. SEMA demonstrated neuroprotective effects, lowering the risk of AD compared to various treatments.

**Conclusion:**

Our systematic appraisal highlighted high heterogeneity across available original investigations. Within this context, meta‐analytic findings suggest that SEMA may be able to promote VAT loss and support cognitive preservation. Sequencing these effects, VAT loss and cognitive preservation, is an important question open for further exploration.

**Abbreviations**
AD: Alzheimer's disease
BIA: bioelectrical impedance analysis
CT: computed tomography
CI: confidence interval
DF: degree of freedom
DEXA: dual x‐ray absorptiometry
ES: effect size
EHR: electronic health records
ETD: estimated treatment difference
ETR: estimated treatment ratio
HR: hazard ratio
LSG: laparoscopic sleeve gastrectomy
LE: limit estimate
MRI: magnetic resonance imaging
NAFLD: nonalcoholic fatty liver disease
PCOS: polycystic ovary syndrome
PRISMA: Preferred Reported Items for Systematic Review and Meta‐Analysis
SEMA: semaglutide
SD: standard deviation
SE: standard error
SMD: standardized mean difference
SAT: subcutaneous adipose tissue
T2DM: type 2 diabetes mellitus
VAT: visceral adipose tissue

## 1 Introduction

Adiposity measurements, mainly visceral adipose tissue (VAT) and subcutaneous adipose tissue (SAT), have demonstrated superiority over body mass index (BMI) in distinguishing between fat and lean mass, representing body fat composition, and predicting abdominal fat distribution by age and sex [1, 2, 3]. Additionally, studies link midlife obesity and adiposity measures, particularly VAT and VAT/SAT ratio, with the pathological signs and incidence of mild cognitive impairment (MCI) and Alzheimer's disease (AD) [4, 5, 6, 7]. Mechanistically, visceral adiposity drives the intersection of obesity, diabetes, and AD by paving the way for the hyper‐release of glucose‐regulatory hormones, incretins, and inflammatory cytokines [8, 9, 10].

Accordingly, interventions targeting the adipose‐cognitive connection are of great promise in treating obesity and diabetes and preventing MCI/AD. Semaglutide (SEMA) is a glucagon‐like peptide‐1 receptor agonist (GLP‐1 RA) recently (in 2019) approved by the US Food and Drug Administration for weight management in people with obesity aged 12 years and older. Studies have frequently shown significant changes in VAT/SAT [11, 12, 13, 14, 15, 16, 17, 18, 19, 20, 21, 22, 23, 24, 25, 26, 27, 28], as well as in the incidence of cognitive issues with SEMA [29, 30, 31, 32].

The present meta‐analysis aims to answer how SEMA influences adiposity and cognition by systematically reviewing human clinical studies that have assessed the effects of SEMA on one or more of the following three domains: (A) the adipose tissue, both VAT and SAT, as assessed by bioelectrical impedance analysis (BIA), dual x‐ray absorptiometry (DEXA), computed tomography (CT), magnetic resonance imaging (MRI), or ultrasound; (B) the brain function as assessed by MRI; and (C) the cognitive performance, as assessed on objective cognitive tasks, or by the incidence of dementia.

## 2 Methods

The present study follows the Preferred Reported Items for Systematic Review and Meta‐Analysis (PRISMA) statement, which was first published in 2009 [33] and updated in 2021 (Supplemental File 1) [34].

### 2.1 Search Strategy

We searched the databases PubMed, Scopus, Web of Science, and EMBASE on March 27, 2023, using the queries detailed in Supplemental File 2. There was no restriction on the publication date. The search results were limited to the English language. We updated the search on November 9, 2024, using PubMed and Scopus. All search records were imported into reference management software. After removing duplicates, automatically and manually, the records were screened based on title and abstract. Irrelevant articles were excluded, whereas the potentially eligible records remained for a detailed review of the full text against the selection criteria. In addition to databases, the references cited in the relevant studies were checked to identify any additional studies missed during electronic database searches.

### 2.2 Selection Criteria

Articles were eligible for inclusion if they met the following criteria:

- They were original research studies, randomized controlled trials (RCTs), nonrandomized controlled trials, uncontrolled single‐group pre‐post studies, or case series.
- They administered SEMA treatment, either as an add‐on or standalone, to participants with any clinical diagnosis.
- They assessed outcomes of interest discussed in subsection 2.3 below
- They reported evaluating outcomes before and after treatment. This should at least apply to the group assigned to the SEMA treatment, as required for inclusion in meta‐analyses of pre‐post, one‐group comparisons. Preferably, if available in RCTs, this should include other treatment arms for meta‐analyses comparing pre‐post, two‐group outcomes (SEMA vs. placebo or another treatment).

Studies used SEMA for both weight loss and T2D management purposes. As a result, the populations varied, with some studies specifically targeting individuals with T2D, while others included individuals focused primarily on weight loss.

Studies were included if they specifically investigated the impact of SEMA as a treatment. Any studies that presented data on SEMA as part of the broader category of drugs, such as GLP‐1 receptor agonists (GLP‐1DAs) or incretin mimetics, without providing specific data for the SEMA subgroup were excluded. This exclusion was necessary to maintain consistency in the type of intervention analyzed and to focus on outcomes related explicitly to SEMA. While the primary emphasis of this study is on SEMA, comparisons with GLP‐1DAs and incretin mimetics were included in order to provide context and assess the relative impact of SEMA compared to the general effects of these other treatments.

Animal, in vivo, in vitro, and in silico studies were excluded. Articles that investigated the economic and financial aspects of SEMA as a treatment, including cost‐effectiveness, pricing, and preference analysis, were also not eligible. Conference or meeting abstracts, non‐English publications, and nonoriginal articles, such as reviews (both narrative and systematic) and meta‐analyses, were also excluded. When more than one publication related to the same original study was available, the most recent version was selected.

### 2.3 Outcomes

The outcomes of interest included measures of (A) SAT and VAT, assessed using BIA, CT, DEXA, MRI, or ultrasound; (B) brain MRI; and (C) performance on objective cognitive tasks (related to memory, executive function, problem‐solving, learning, impulsivity, information processing, and attention), and incidence of cognitive impairment and dementia.

The analysis was planned to run over pre‐post one‐group (SEMA) and two‐group (SEMA vs. comparative interventions, for example, placebo, control, or other therapeutics) comparisons, considering the possible contribution of all individual studies to the summary effect size (ES) with any measure made for the outcomes of interest. In studies that provided assessments for different time points, we chose the most commonly assessed time point across studies entered into the meta‐analysis to avoid the inclusion of the same study more than once in the same analysis.

Our scoping search yielded an insufficient number of studies to perform a meta‐analysis on the effects of SEMA on brain and cognitive function. Consequently, these studies will be included exclusively in a qualitative synthesis. The subsequent sections of the methods will be dedicated to the analysis of SAT/VAT‐related data, which will be incorporated into the quantitative synthesis.

### 2.4 Data Extraction

An Excel worksheet was developed for extracting data on the following items from each included study: (1) study characteristics, including the first author's name, year of publication, and location; (2) methodological characteristics, for example, study design and study quality; (3) intervention characteristics, including treatment arms and dose and duration; (4) population characteristics, for example, the diagnosed condition, number of participants entered into and completed the trial, age, male percentage, and body mass index; and (5) outcome characteristics, that is, the technology and measure(s) used for outcome assessment, time point(s) of outcome assessment, and statistics, for example, mean and standard deviation (SD), standard error (SE), 95% confidence interval (CI), estimated treatment difference (ETD) or ratio (ETR), and *p* value, provided for the measured outcome(s) before and after treatment at each time point for all treatment arms. All the statistics provided in the main manuscript and related supplements, along with the information embedded in the figures, were extracted in order to enable us to calculate effect size. In addition, we emailed the corresponding authors requesting additional data on all studies.

### 2.5 Effect Sizes

We divided the raw mean difference, which is the product of the subtraction of the pre‐treatment and posttreatment mean values, by the SD of change to calculate the standardized difference in means (SMD) as the effect size (ES) measure. To account for variability among pretreatment and posttreatment scores, the SE of the effect size was also estimated, and this required the pre‐post correlation coefficient value, which represents the relationship between measured outcomes at the tested time points. When sufficient data were available, we calculated the pre‐post correlation coefficient according to the Cochrane guideline [35]; otherwise, we proceeded with the imputation of a value of 0.6 [36]. Dealing with studies with missed posttreatment SD or SD of change but provided ETD and 95% CI, we obtained SMD with the method mentioned above and ES directly from 95% CI [35].

The above data imputation and harmonization procedure was performed on the data from one group (SEMA) or from two groups (SEMA compared to another treatment), depending on the study design and data availability. In all analyses, a negative SMD would represent a favorable event that occurred in the outcome of interest following SEMA treatment. For example, in one‐group analyses, it was simply VAT reduction after SEMA treatment compared to before treatment; in two‐group analyses, it was the improvement of VAT reduction by SEMA treatment in comparison with placebo or another treatment.

### 2.6 Data Analysis

The ReviewManager (RevMan) Version 5 was used for the meta‐analysis. We used the generic inverse variance outcome type and directly entered SMDs and related SEs to obtain the summary effect size and related 95% CI and *p* value under the random effects model. This analysis also provided information about the presence and magnitude of heterogeneity across studies in terms of Q and *I* ^2^ statistics; however, these available tests were not reliable for interpretation when there were fewer than 10 studies. The ES's magnitude ranges from small to medium to large, as defined by SMD cutoffs of 0.2, 0.5, and 0.8. A meta‐analysis was performed whenever three or more relevant observations on the topic were available.

### 2.7 Additional Analyses

Leave‐one‐out sensitivity analyses were done for all meta‐analyses to determine if any study drives the results or if they are robust across studies. In addition, whenever the number of observations included in a meta‐analysis was suitable (≥ 10), we explored the possible sources of heterogeneity by subgroup and regression analyses, as well as running the tests for investigating the presence of publication bias. These analyses were done in R‐4.4.2 using packages for meta‐analysis, including “metafor.”

### 2.8 Quality Assessment

The quality of the studies was appraised based on the Guidelines for Critical Review of Quantitative Studies developed by Law et al. in 1998 [37]. With this tool, each article is assessed for eight domains: study purpose, literature review, design, sample, outcomes, intervention, results, and conclusions and implications. There is no consistent cutoff for defining a study of an acceptable quality. Systematic reviews have considered scores of 8–10 for this purpose [38, 39]. In the present review, the quality score of 10 was the cutoff set up for the inclusion of studies.

### 2.9 Declaration of Generative AI and AI‐Assisted Technologies in the Writing Process

Chat GPT, GPT4, AI, and similar tools were used to refine the language. The content generated in this way was then carefully reviewed and edited to ensure accuracy and readability.

## 3 Results

### 3.1 Study Selection

**FIGURE 1:**
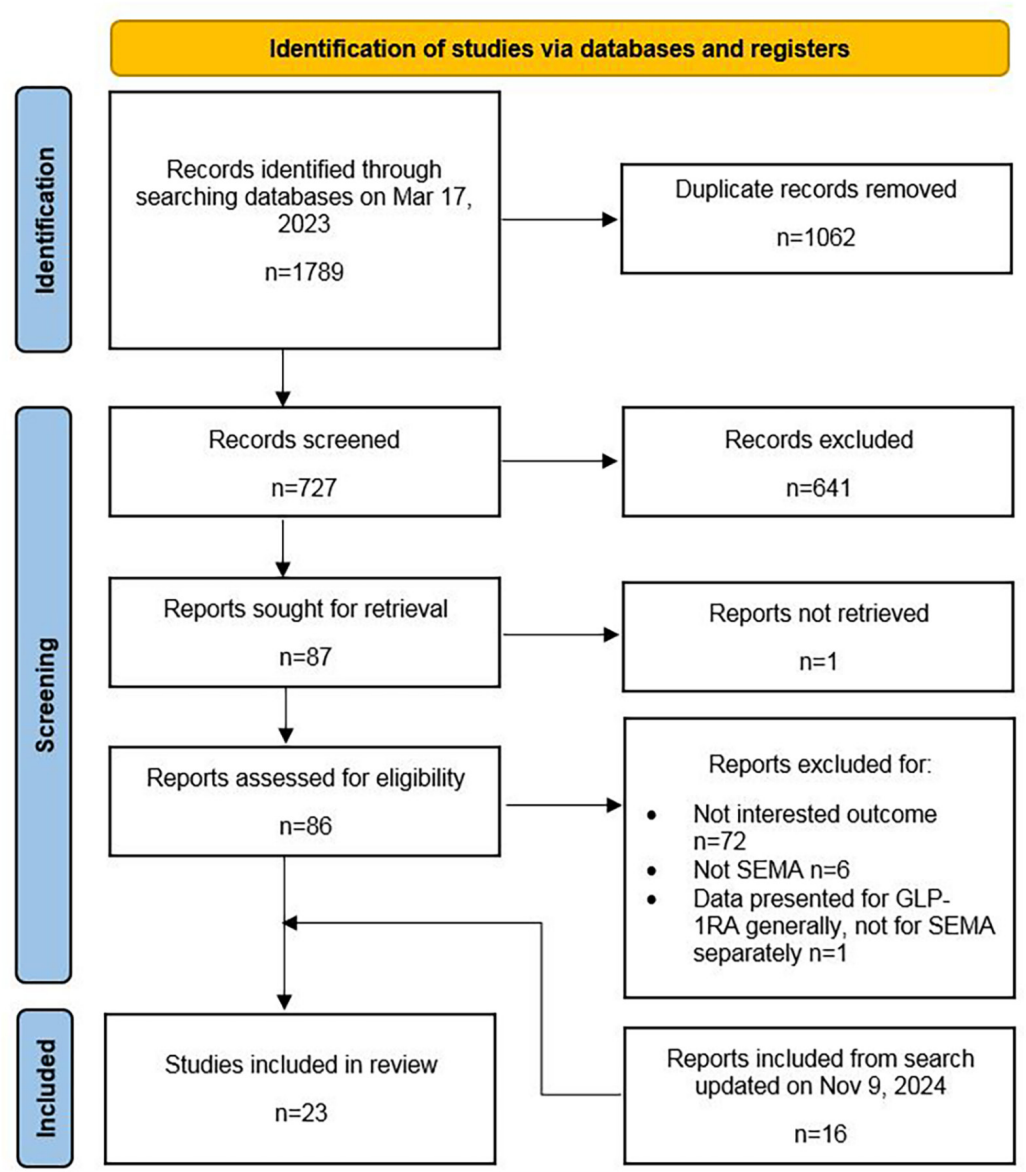
Selection's flow diagram prepared using the PRISMA 2020 flow diagram template.

As depicted in Figure 1, 1789 records were identified by searching databases. After duplicate removal, 727 discrete results remained for screening, through which 641 records were not eligible based on title/abstract. Therefore, we selected 87 studies to retrieve for review; however, the full text of one of them was not available [40], so we proceeded with a detailed eligibility assessment of 86 studies. When reviewing the reports, we had to exclude 79 studies due to inappropriateness with the outcome (*n* = 72), intervention (*n* = 6), or data (data presented for GLP‐1RA generally, not for SEMA specifically) (*n* = 1). The November 2024 search retrieved an additional 16 studies meeting the criteria for inclusion. Finally, 23 studies were included [11, 12, 13, 14, 15, 16, 17, 18, 19, 20, 21, 22, 23, 24, 25, 26, 27, 28, 29, 30, 31, 32, 41].

### 3.2 Study Characteristics

An overview of the characteristics of the studies is presented in Table 1.

**TABLE 1:** Overview of the studies included in the systematic review.

First author, year of publication (reference) Location	Design	Treatment arms	Groups*					Outcome of interest	Outcome assessment	
			Condition	*n* (completed)	Age	%Male	BMI		Method	Time
Flint et al. 2021 [29] Germany/Denmark	Randomized, controlled trial	Subcutaneous SEMA 0.4 mg daily vs. placebo	NAFLD	34 (27) vs. 33 (30)	59.5 (10.1) vs. 60.5 (8.5)	67.6 vs. 72.7	25–40	Visceral and abdominal subcutaneous adipose tissue volume	MRI	Week 24, 48, and 72
Jensterle et al. 2021 [1] Slovenia	Randomized single‐blind, pilot study	SEMA up to 1 mg once weekly vs. placebo	PCOS	15 (13) vs. 15 (12)	33.7 (5.3)	0	36.8 (3.9) vs. 35.4 (3.8)	Visceral adipose tissue mass, volume, and area	DEXA	Week 16
Kadowaki et al. 2022 [2] Japan and South Korea	Randomized, double‐blind, double‐dummy, placebo‐controlled trial STEP 6	SEMA 2·4 mg once weekly vs. SEMA 1·7 mg once weekly vs. placebo	Overweight or obesity	89 (87) vs. 46 (45) vs. 45 (43)	52 (12) vs. 51 (10) vs. 50 (9)	57 vs. 63 vs. 75	32 (4.6) vs. 31.6 (3.7) vs. 31.9 (4.2)	Abdominal visceral fat area	CT	Week 68
McCrimmon et al. 2020 [3] 111 centers in 11 countries	Randomized double‐blind trial SUSTAIN 8	SEMA up to 1 mg once weekly vs. canagliflozin 300 mg once daily	T2DM	88 (76) vs. 90 (76)	57.8 (9.9) vs. 58.6 (10.1)	56.6 vs. 51 vs. 53.8	32.6 (6.4) vs. 32.3 (5.5)	Visceral fat mass	DEXA	Week 52
Volpe et al. 2022 [4] Italy	Prospective, single‐arm, real‐life study	SEMA up to 1 mg once weekly	T2DM	48	57.7 (8.4)	54.2	38.8 (8.3)	Subcutaneous and visceral adipose tissue thickness	Ultrasound	Month 3, 6, and 12
Volpe et al. 2022 [5] Italy	Prospective, single‐arm, real‐life study	Subcutaneous SEMA 1 mg once weekly	T2DM	40	Not mentioned	52.5	38.8 (7.7)	Visceral adipose tissue thickness and volume	BIA and ultrasound	Month 3 and 6
Volpe et al. 2023 [6] Italy	Prospective, single‐arm, real‐life study	Subcutaneous SEMA 1 mg once weekly	T2DM	32	66.3 (8.5)	56.7	28.2 (3.3)	Visceral adipose tissue volume	BIA	Month 3 and 6
Wilding et al. 2021 [7] 129 sites in 16 countries	Double‐blind trial STEP 1	SEMA 2·4 mg once weekly vs. placebo	Overweight or obesity	95 vs. 45	50 (12) vs. 52 (13)	24.2 vs. 24.4	34.8 (3.6) vs. 35 (3.6)	Visceral fat mass	DEXA	Week 68
Cirnigliaro et al. 2024 [8] USA	Open‐label controlled case series	Subcutaneous SEMA 1.0 mg weekly vs. no treatment	Wheelchair‐dependent participants with SCI	3 vs. 2	47 (11) vs. 53 (4)	100% vs. 100%	29.5 (1.2) vs. 32.6 (4.1)	Visceral adipose tissue volume	iDXA	Week 26
Dusilová et al. 2024 [9] Czech Republic	Cross‐over study without a washout period	Dietary intervention and treatment with subcutaneous SEMA 1.0 mg weekly	Obesity	16	47 (12.4)	100%	36.2 (3.8)	Subcutaneous fat area and visceral fat area	MRI	Week 14–16 and 30–32
Eckard et al. 2024 [10] USA	Randomized, double‐blind, placebo‐controlled Phase 2b single‐center clinical trial	Subcutaneous SEMA 1.0 mg weekly vs. placebo	HIV‐associated lipohypertrophy	54 (46) vs. 54 (46)	53 (40 to 57) vs. 53 (41 to 57)	70% vs. 50%	32·9 (28·4 to 36·0) vs. 33·8 (29·9 to 39·7)	Subcutaneous and visceral adipose tissue area and density	CT	Week 32
García‐Vega et al. 2024 [11] Spain	Observational and longitudinal study	Subcutaneous SEMA 0.5–1.0 mg weekly	Diabetes mellitus (T2DM), BMI ≥ 30 kg/m^2^ and with poor glycemic control (Hb1AC ≥ 7%), despite two antidiabetic drugs	20 (19)	63.6 (11.9)	80%	37.2 (5.8)	Visceral adipose tissue area	BIA	Month 6
Ingersen et al. 2023 [12] Denmark	Randomized clinical trial	Subcutaneous SEMA 1.0 mg weekly and training vs. training	Diabetes mellitus (T2DM) and overweight or obesity	16 vs. 15	59 (6.2) vs. 56 (5.7)	87.5% vs. 86.7%	34.8 (4.8) vs. 34.6 (5.4)	Visceral fat mass	Not mentioned	Week 12, 20, and 32
Kanai et al. 2024 [13] Japan	Single‐center retrospective database analysis	SEMA 0.25–1.0 mg weekly	T2D who underwent LSG, and more than 12 months later received SEMA	29	47.2 (8.7)	58.6%	46.5 (11.0)	Subcutaneous and visceral adipose tissue area	CT	Month 12
Nelson et al. 2024 [14] USA	Single‐center, HIPAA‐compliant, retrospective study	SEMA	Adult patients with SEMA treatment who underwent abdominopelvic CT both within 5 years before and within 5 years after SEMA initiation	241	60.5 (12.4)	37.3%	38.7	Subcutaneous and visceral adipose tissue area and attenuation	CT	Within 5 years
Rodríguez Jiménez et al. 2024 [15] Spain	Quasi‐experimental retrospective study	Subcutaneous SEMA 1.0 mg weekly vs. oral SEMA 14.0 mg daily	T2DM and obesity with BMI ≥ 30 kg/m^2^	55 vs. 33	55.3 (10.4) vs. 61.8 (7)	52.7% vs. 66.7%	40.1 (11) vs. 33.2 (3.9)	Visceral adipose tissue area	BIA	Month 6
Stafeev et al. 2024 [16] Russia	Observational retrospective study	Subcutaneous SEMA 1.0 mg weekly	T2DM and long‐ morbid (> 10 years) obesity (BMI > 35 kg/m^2^)	8	62 (55.8–65)	50%	39.4 (38.2–43.6)	Visceral adipose tissue area	BIA	Month 6
Song et al. 2024 [17] China	Observational retrospective study	Subcutaneous SEMA 1.0 mg weekly	People with overweight or obesity and without diabetes	84	33.8 (7.6)	11.9%	28.5 (4.4)	Visceral adipose tissue area	BIA	Week 12
Verovnik et al. 2024 [18] Slovenia	Derived from a randomized, single‐blind, placebo‐controlled study	Subcutaneous SEMA 1.0 mg weekly vs. placebo	Women ith obesity and polycystic ovary syndrome	10 vs. 8	31.2 vs. 34.0	0	Not mentioned	Resting state functional connectivity	MRI	Week 16
Siddeeque et al. 2024 [19] 17 countries	Retrospective propensity‐score matched cohort study	Semaglutide vs. none	Obese adult patients	57,043 vs. 57,043	50.6 (14.4) vs. 50.6 (14.4)	31.5 vs. 33	Not mentioned	Risk of AD, Lewy body dementia, and vascular dementia	Exploring a global collaborative network spanning 17 countries and 152,398,854 patients as of July 31, 2024	Not specified
		Dulaglutide vs. none		19,165 vs. 19,165						
		Liraglutide vs. none		9724 vs. 9724						
Wadden et al. 2024 [20] Multicenter	Data from the randomized, double‐blind, placebo‐controlled, multicenter Phase 3a STEP 1, 2, and 3 trials	Subcutaneous SEMA 2.4 mg vs. placebo	Adults with overweight or obesity without known major psychopathology	2116 vs. 1261	48 (13) vs. 50 (13)	29.5 vs. 31.2	37.5 (6.7) vs. 37.3 (6.7)	Risk of cognitive and attention disorders and disturbances	Post hoc analysis	Week 68
	Data from Phase 3b STEP 5 trial			152 vs. 152	47 (12) vs. 47 (10)	19.1 vs. 25.7	38.6 (6.7) vs. 38.5 (7.2)			
Wang et al. 2024 [21] USA	Emulation target trials	SEMA vs. insulin	Patients withT2DM who had no prior AD diagnosis	17,087 vs. 17,087	58.1 (12.1) vs. 58.0 (14.8)	40.4 vs. 39.8	Not mentioned	First‐ever diagnosis of AD	Exploring a nationwide database of electronic health records of 116 million US patients	Within 3 years
		SEMA vs. metformin		17,080 vs. 17,080	Not mentioned					
		SEMA vs. DPP‐4i		15,878 vs. 15,878						
		SEMA vs. SGLT2i		15,288 vs. 15,288						
		SEMA vs. SU		16,503 vs. 16,503						
		SEMA vs. TZD		10,487 vs. 10,487						
		SEMA vs. other GLP‐1RAs		17,029 vs. 17,029						
De Giorgi et al. 2024 [22] USA	Retrospective propensity‐score matched cohort study	SEMA vs. sitagliptin	T2DM	23,386 vs. 23,386	56.7 (12.2) vs. 56.6 (13.3)	43.5 vs. 43.9	Not mentioned	First diagnosis for cognitive deficit and dementia	Exploring electronic health records from TriNetX US Collaborative Network, covering > 100 million patients in the USA	Within the year
		SEMA vs. empagliflozin		22,584 vs. 22,584	57.6 (12.3) vs. 57.6 (12.4)	42.7 vs. 42.9				
		SEMA vs. glipizide		19,206 vs. 19,206	56.4 (12.4) vs. 56.2 (13.6)	42.4 vs. 42.2				

* Characteristics of groups are preferrably related to the subset employed as the sample for the comparison included in the review, but when data were not available, we relied on the information related to the whole population of the original trials.

Abbreviations: AD, Alzheimer's diseases; BMI, body mass index; CT, computed tomography; DEXA, dual x‐ray absorptiometry; MRI, magnetic resonance imaging; *n*, number; NA, not addressed; NAFLD, nonalcoholic fatty liver disease; PCOS, polycystic ovary syndrome; SCI, spinal cord injury; SEMA, semaglutide; T2DM, type 2 diabetes mellitus.

#### 3.2.1 SEMA and Adipose Tissue

Eighteen of 23 studies measured at least one of the outcomes of interest for SAT and VAT [11, 12, 13, 14, 15, 16, 17, 18, 19, 20, 21, 22, 23, 24, 25, 26, 27, 28].

VAT‐related indices were assessed in 18 studies, including area in 10 studies [12, 13, 15, 17, 18, 19, 21, 22, 23, 24], attenuation in one study [21], density in one study [13], mass in four studies [16, 17, 20, 28], thickness in two studies [26, 27], and volume in five studies [14, 17, 25, 26, 27].

Six studies assessed SAT‐related indices: area in four studies [12, 13, 19, 21], attenuation in one study [21], density in one study [13], thickness in one study [26], and volume in one study [14].

Seventeen out of 18 studies (> 94%) provided details on the dose, route, schedule, and duration of SEMA treatment. Among these studies, 15 administered SEMA through subcutaneous injections [11, 12, 13, 14, 15, 16, 17, 18, 19, 20, 23, 24, 26, 27, 28], one study compared oral and subcutaneous SEMA therapy across two arms [22], and another study solely used oral SEMA [25]. In the studies utilizing oral SEMA, a daily dosage of up to 14 mg was used [22, 25]. For subcutaneous SEMA therapy, all but one study employed a weekly administration schedule; one study instead used a daily dose of 0.4 mg [14].

Among the 15 studies that utilized weekly subcutaneous SEMA therapy, 13 increased the dosage to 1 mg [11, 12, 13, 15, 16, 17, 19, 20, 22, 23, 24, 26, 27]. One study involved two arms with different dosing schedules, raising the doses to a maximum of 1.7–2.4 mg [18], while another study increased the dosage to 2.4 mg [28].

Some studies reported durations in months. We calculated the duration of SEMA therapy by considering each month to consist of 4 weeks. As a result, the duration ranged from 12 weeks to 72 weeks, with a median of 24 and a mean of 34.7 (SD of 18.8 weeks).

Only one study, which involved a retrospective analysis of electronic medical records, lacked specific information regarding the SEMA therapy [21].

All studies provided information on age, percentage of males, and BMI for participants enrolled in SEMA therapy. However, two studies did not include age data [17, 27], one study lacked a percentage of males [20], and another was missing BMI information [14]. Among the studies that included sufficient data for individuals treated with SEMA, the average age ranged from 33.9 to 66.3 years, with a median of 56 years and a mean of 54.3 years (SD of 7.6 years). The percentage of males varied from 11.9% to 100%, with a median of 58.6% and a mean of 62.8% (SD of 23.9%). The average BMI ranged from 24.5 to 40.4 kg/m^2^, with a median of 34.8 and a mean of 34.7 (SD of 4.1 kg/m^2^).

Participants enrolled in SEMA therapy, in addition to being overweight or obese and having or not having diabetes, had specific conditions in certain studies. These conditions included having undergone laparoscopic sleeve gastrectomy (LSG) 12 months prior to starting SEMA therapy [19], polycystic ovary syndrome (PCOS) [17], nonalcoholic fatty liver disease (NAFLD) [14], chronic spinal cord injury (SCI) [11], and HIV‐associated lipohypertrophy [13].

Techniques for measuring SAT/VAT‐related indices included BIA in five studies (27.8%) [15, 22, 23, 24, 25], BIA and ultrasound in two studies (11.1%) [26, 27], CT in four studies (22.2%) [13, 18, 19, 21], DEXA in five studies (27.8%) [11, 16, 17, 20, 28], and MRI in two studies (11.1%) [12, 14].

Ten studies evaluated the effects of SEMA alone (55.6%) [12, 15, 19, 21, 22, 23, 24, 25, 26, 27], while eight (44.4%) included at least one other comparative intervention arm, such as placebo, dietary interventions, training, and other therapeutic agents [11, 13, 14, 16, 17, 18, 20, 28].

Studies were designed as follows: seven studies (38.9%) were experimental randomized controlled trials (RCTs) [13, 14, 16, 17, 18, 20, 28], one study (5.6%) was an experimental cross‐over design [12], one study (5.6%) was quasi‐experimental [22], eight studies (44.4%) were observational (encompassing retrospective, prospective, and mixed/other designs) [15, 19, 21, 23, 24, 25, 26, 27], and one study (5.6%) was a case series [11].

A total of 1224 participants were included in the SEMA therapy arms, with a median of 37 participants across the studies.

#### 3.2.2 SEMA and Brain Function

We identified a study that examined the brain's response to SEMA using MRI [41]. It was a 16‐week RCT of women with obesity and PCOS, monitoring the resting state functional connectivity (RSFC) in specific regions of interest (ROIs) related to suicidal ideation and behavior in major depressive disorder. A total of 18 participants were included in the analysis.

#### 3.2.3 SEMA and Cognitive Function

Evidence addressing the impact of SEMA on cognitive performance was found in four studies [29, 30, 31, 32].

The first study was a retrospective propensity score‐matched (PSM) cohort analysis that determined the risk of being diagnosed for the first time with cognitive deficits and dementia in patients with T2DM. This research utilized electronic health records (EHRs) from the TriNetX US Collaborative Network, which encompasses over 100 million patients in the USA. A total of 65,176 participants were included in the analysis [30].

The second study also used a retrospective PSM cohort design to assess the risk of AD, Lewy body dementia, and vascular dementia in adults with obesity. This research was conducted within a global collaborative network covering 17 countries, with 152,398,854 patients as of July 31, 2024. The cohort included 102,935 patients [29].

The third study performed a post hoc analysis of data from three Phase 3a RCTs—STEP 1, 2, and 3—and one Phase 3b RCT, STEP 5. This study estimated the risk of cognitive and attention disorders in adults with overweight or obesity who did not have known major psychopathology. The analysis encompassed 3681 participants [31].

The fourth and most recent study was the largest retrospective PSM cohort investigation. It determined the risk of a first‐time diagnosis of AD in patients with T2DM by analyzing EHR data from 116 million US patients. A total of 1,094,761 patients were included in this analysis [32].

Additionally, there was a study [42] that pooled data on dementia incidence in patients treated with GLP‐1 receptor agonists (RAs) who had T2DM. This study included three large‐scale trials that focused on the cardiovascular effects of GLP‐1 RAs and calculated a hazard ratio of 0.89 (95% confidence interval: 0.86–0.93) for dementia incidence. SEMA was the intervention of interest in two of these trials [43, 44], while Liraglutide was used in another [45]. Therefore, based on the pooled data, it is difficult to conclude whether GLP‐1 RAs have a beneficial effect on reducing dementia incidence due to SEMA's contribution or to determine SEMA's net impact in this regard.

### 3.3 Meta‐Analyses of the Effect of SEMA on VAT Area

#### 3.3.1 Pre‐Post SEMA

**FIGURE 2:**
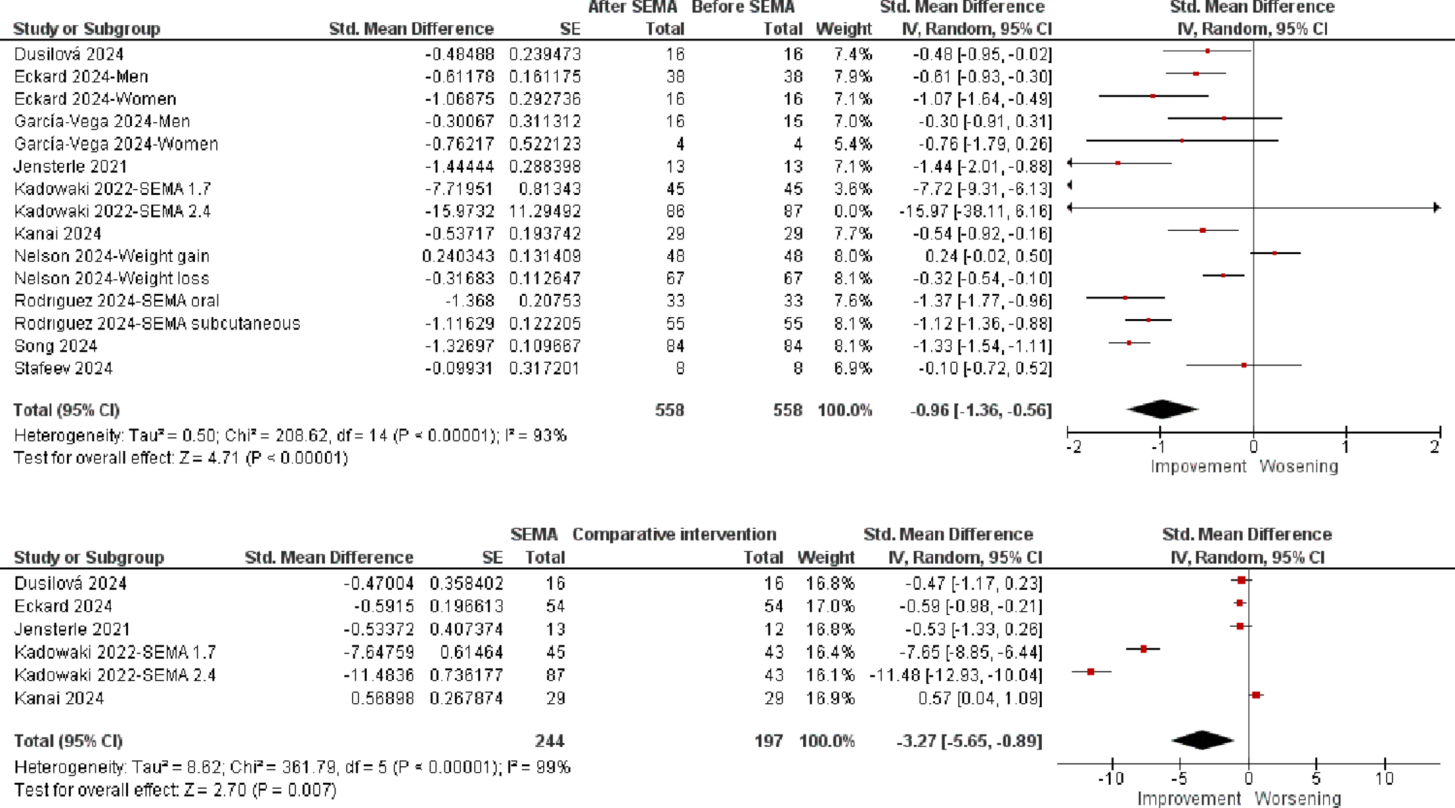
Forest plots of effects of SEMA on VAT area.

A total of 15 observations from 10 studies, including 558 patients, were put into the meta‐analysis of the pre‐post effect of SEMA on the VAT area. An overall effect size of −0.96 (0.95% CI: −1.36–0.56; Z = 4.71, *p* < 0.00001, Table 2) revealed a significant, large VAT area reduction by SEMA (Figure 2, top).

**TABLE 2:** Meta‐analyses of SEMA effect on SAT and VAT.

Outcome	Comparison	Number of observations	Effect size	95% confidence interval	*Z*	*p*
**VAT mass**	Post‐SEMA vs. pre‐SEMA	4	−0.91	−1.36 to −0.45	3.92	< 0.0001
	SEMA vs. comparative intervention (pre‐post comparison)	4	−0.25	−0.46 to −0.04	2.30	0.02
**VAT volume**	Post‐SEMA vs. pre‐SEMA	3	−1.43	−2.38 to 0–0.47	2.94	0.003
	SEMA vs. comparative intervention (pre‐post comparison)	3	−0.39	−1.04 to 0.26	1.17	0.24
**VAT area**	SEMA vs. comparative intervention (pre‐post comparison)	6	−3.27	−5.65 to −0.89	2.70	0.007
	Post‐SEMA vs. pre‐SEMA	15	−0.96	−1.36 to −0.56	4.71	< 0.00001
**SAT area**	Post‐SEMA vs. pre‐SEMA	6	−0.35	−0.71 to 0.01	1.90	0.06
	SEMA vs. comparative intervention (pre‐post comparison)	3	−0.45	−1.45 to 0.54	0.89	0.37

Abbreviations: SAT, subcutaneous adipose tissue; SEMA, semaglutide; SMD, standardized mean difference; VAT, visceral adipose tissue.

##### 3.3.1.1 Sensitivity Analyses

The sensitivity analysis (excluding observations one‐by‐one) revealed that the *p* values remained highly significant (< 0.00001 to < 0.0001) for all exclusions, with effect sizes ranging from 4.19 to 5.38. There were two extreme outliers (SMD = −7.72 and SMD = −15.97), but their exclusion did not significantly alter the significance or effect size of the meta‐analysis. The *I* ^2^ values remained high (90%–94%) and did not substantially change when any single study was excluded.

##### 3.3.1.2 Subgroup Analyses

Subgroup analyses were conducted to assess whether the study design and measurement techniques contributed to the overall heterogeneity of the findings (*I* ^2^ = 93%).

In the overall analysis, the following 15 observations were included: seven from observational studies, five from RCTs, two from a quasi‐experimental study, and one from a cross‐over study (Table 3). The cross‐over and quasi‐experimental subgroups exhibited a low *I* ^2^ value of 0%–8%, and the observational and RCT subgroups exhibited high *I* ^2^ values of 94%–95%. The effect size for the observational subgroup was not statistically significant (*p* = 0.098), and the cross‐over subgroup's effect size was marginal (*p* = 0.043), with small magnitudes of −0.44 and −0.48, respectively. Conversely, the RCT and quasi‐experimental subgroups showed highly significant effects (*p* values of 0.0009 and < 0.000001), with large magnitudes of −2.48 and −1.19.

**TABLE 3:** Subgroup analyses of SEMA effect on VAT area in single‐group pre‐post comparisons.

Moderator	Subgroup	Number of observations	Effect size	*p*	95% confidence interval	*Z*	*I* ^2^%	Observations
**Study design**	Observational	7	−0.44	0.098	−0.96 to 0.08	−1.65	94	Song 2024–SEMA, García‐Vega 2024–Men, García‐Vega 2024–Women, Stafeev 2024–SEMA, Kanai 2024–SEMA, Nelson 2024–Weight gain, Nelson 2024–Weight loss
	Cross‐over study	1	−0.48	0.043	−0.95 to −0.02	−2.02	0	Dusilová 2024–SEMA
	RCT	5	−2.48	0.0009	−3.94 to −1.01	−3.31	95	Jensterle 2021–SEMA, Eckard 2024–Men, Eckard 2024–Women, Kadowaki 2022–SEMA1.7, Kadowaki 2022–SEMA2.4
	Quasi‐experimental	2	−1.19	< 0.00001	−1.41 to −0.97	−10.52	8	Rodrıguez 2024–Oral SEMA, Rodrıguez 2024–Subcutaneous SEMA
**Measurement technique**	BIA	6	−0.92	< 0.00001	−1.28 to −0.57	−5.13	78	Song 2024–SEMA, García‐Vega 2024–Men, García‐Vega 2024–Women, Rodrıguez 2024–Oral SEMA, Rodrıguez 2024–Subcutaneous SEMA, Stafeev 2024–SEMA
	MRI	1	−0.48	0.043	−0.95 to −0.02	−2.02	0	Dusilová 2024–SEMA
	DEXA	1	−1.44	< 0.00001	−2.01 to −0.88	−5.01	0	Jensterle 2021–SEMA
	CT	7	−1.16	0.001	−1.86 to −0.46	−3.24	95	Eckard 2024–Men, Eckard 2024–Women, Kanai 2024‐–SEMA, Kadowaki 2022–SEMA1.7, Kadowaki 2022–SEMA2.4, Nelson 2024–Weight gain, Nelson 2024–Weight loss

Among the 15 observations in the overall analysis, six were classified in the BIA subgroup, seven in the CT subgroup, one in the MRI subgroup, and one in the DEXA subgroup. Given the small number of observations in the MRI and DEXA subgroups, the finding of low heterogeneity (*I* ^2^ value of 0%) also requires caution. The CT and BIA subgroups, on the other hand, demonstrated high *I* ^2^ values (78%–95%). Regarding *p* values, the most significant effect was observed in the BIA and DEXA subgroups (*p* < 0.00001), followed by the CT subgroup (*p* = 0.001) and the MRI subgroup (*p* = 0.043). In terms of effect size magnitude, the DEXA subgroup had the largest effect (−1.44), followed by the CT subgroup (−1.16), BIA subgroup (−0.92), and MRI subgroup (−0.48).

##### 3.3.1.3 Meta‐Regression

###### 3.3.1.3.1 Univariate Meta‐Regression

Univariate meta‐regressions were run to explore the relationships between potential moderators and the SMD (Table 4). Four continuous moderators, age, BMI, sex, and duration, were considered. For both age and percentage of males, neither the model nor the factor itself was statistically significant, nor did they show a significant relationship with SMD.

**TABLE 4:** Meta‐regression analyses of SEMA effect on VAT area in single‐group pre‐post comparisons.

Moderator	Model results (coefficients)	Estimate	SE	Z	*p*	*I* ^2^%	Overall *R* ^2^
**Age**	Intercept	−3.57	4.31	−0.83	0.408	99	0
	Age	0.04	0.08	0.48	0.629	99	0
**BMI**	Intercept	−8.81	3.93	**−2.24**	**0.025**	99	19.33
	BMI	0.2	0.1	**1.95**	**0.051**	99	19.33
**Sex**	Intercept	−1.45	0.91	−1.59	0.112	99	0
	Male%	0	0.01	0.12	0.906	99	0
**Treatment duration**	Intercept	1.15	0.87	1.31	0.189	97	43.05
	Treatment duration	−0.09	0.03	**−3.12**	**0.002**	97	43.05
**BMI and treatment duration**	Intercept	−7.54	1.98	**−3.81**	**0.0001**	89	86.05
	BMI	0.24	0.06	**4.31**	**0.00002**	89	86.05
	Treatment duration	−0.1	0.02	**−5.3**	**< 0.00001**	89	86.05
**BMI, treatment duration, study design, and measurement technique**	Intercept	−2.76	0.89	**−3.1**	**0.002**	0	100
	BMI	0.04	0.03	1.11	0.27	0	100
	Treatment duration	0.06	0.03	1.66	0.1	0	100
	Design observational	−0.33	0.3	−1.08	0.28	0	100
	Design quasi experimental	−1.22	0.34	**−3.62**	**0.0003**	0	100
	Design RCT	−8.1	1.54	**−5.26**	**< 0.00001**	0	100
	Technique CT	−1.91	0.78	**−2.46**	**0.01**	0	100
	Technique DEXA	7.12	1.57	**4.55**	**< 0.00001**	0	100

**FIGURE 3:**
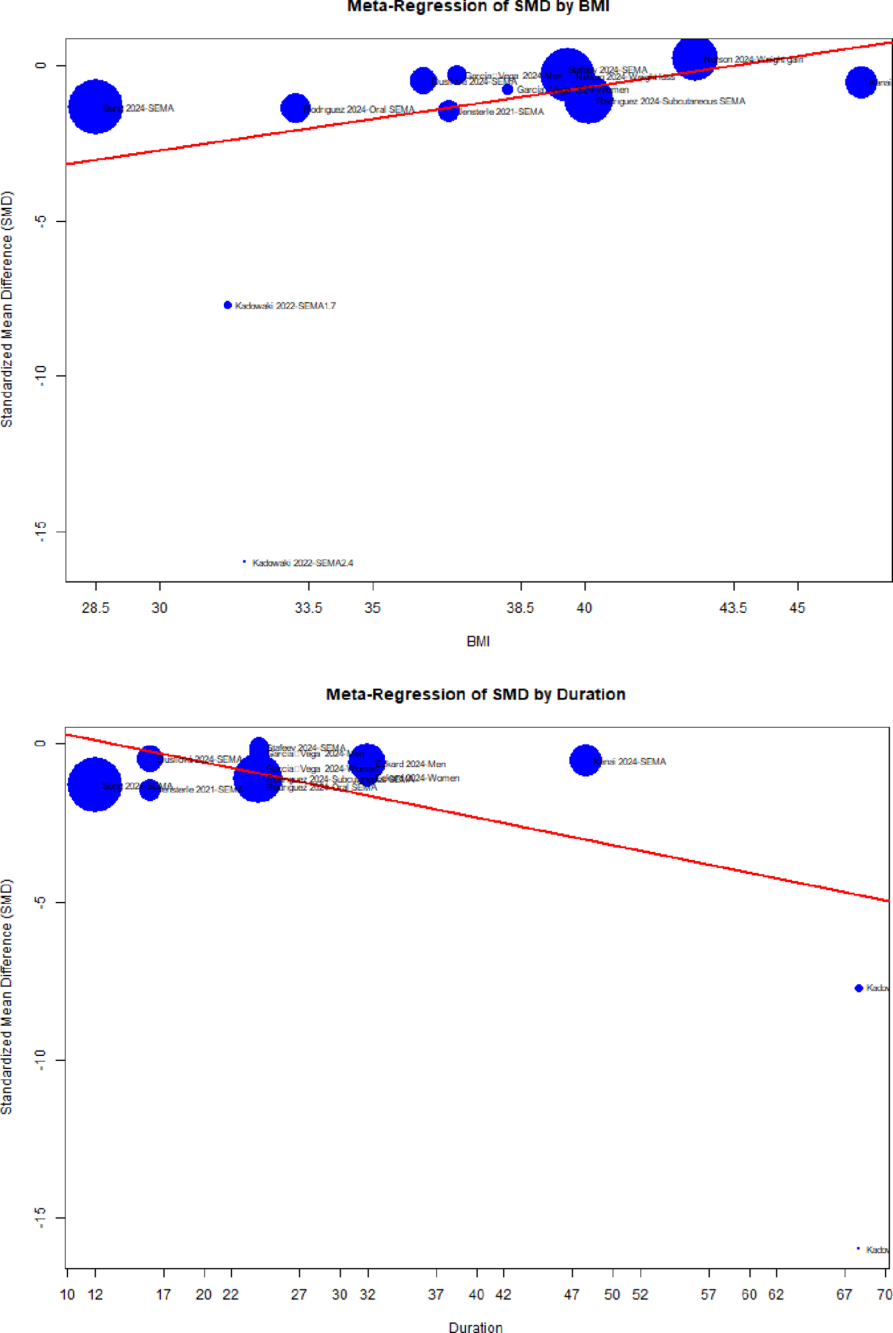
Forest plots of effects of SEMA on VAT mass.

There was a positive relationship between BMI and SMD (coefficient of 0.2), suggesting that as BMI increases, the SMD becomes less negative (Figure 3, top). I.e., for individuals with higher BMI, the VAT area reduction is less pronounced compared to individuals with lower BMI.

Though the overall *p* value for the model (0.025) indicated that the model as a whole is statistically significant, BMI itself was only marginally significant with a *p* value just above the significance level (0.051).

For the duration, the overall model *p* value (0.189) was not significant, but duration itself was a significant moderator (*p* = 0.002). A coefficient of −0.09 means that as duration increases, the SMD becomes more negative (Figure 3, bottom). I.e., for individuals with longer SEMA therapy, the VAT area reduction was more pronounced compared to individuals with shorter SEMA therapy.

The *R* ^2^ values suggest that BMI and duration alone explain about 19.3% and 43.0% of the variation in SMD.

###### 3.3.1.3.2 Multivariate Meta‐Regression

We proceeded with running a multivariate meta‐regression considering BMI and duration as moderators.

Coefficients for the overall model (*p* = 0.0001), as well as BMI (*p* = 0.00002) and duration (*p* < 0.00001), were all statistically significant. Similar to univariate models, BMI and duration showed a significant positive and negative relationship with SMD.

The *R* ^2^ indicated that the model explains 86.05% of the residual variability in SMD. This high proportion suggests that BMI and duration are strong predictors of SMD across studies. The test of moderators resulted in the QM statistic of 34.4 (df = 2) with a *p* value < 0.0001, suggesting that BMI and duration as moderators collectively explain a significant portion of the variability in SMD across studies. In addition, the test for residual heterogeneity yielded the QE statistic of 65.4 (df = 8) with a *p* value < 0.0001, indicating that there is significant residual heterogeneity across studies that remains unexplainable by BMI and duration.

###### 3.3.1.3.3 Mixed‐Effects Meta‐Regression

We ran a mixed‐effects model, comprising BMI, duration, study design, and measurement technique. With this model, there was no residual variability as reflected in tau^2^ of 0 and *I* ^2^ of 0%. *R* ^2^ of 100% suggests that the moderators explain all the variability in the data. The *p* for the QE statistics of 1.6 (df = 3) was high, indicating no evidence of residual heterogeneity after fitting the model. In addition, the *p* for the QM statistic of 105.7 (df = 7) was < 0.001, indicating that the combination of BMI, duration, design, and technique significantly explains the variability in the SMD.

##### 3.3.1.4 Publication bias

Egger's test (Supplemental Figure S1) was significant (Z = −2.16, *p* = 0.031). The limit estimate (LE) model resulted in a b value of −0.758 (with a 95% CI of −1.96 to 0.45). The trim‐and‐fill method identified no missing studies for inclusion and asymmetry correction.

#### 3.3.2 Pre‐Post SEMA vs. Comparative Intervention

There were six comparisons extracted from five eligible studies. The ES of −3.27 was significant and large in magnitude (95% CI: −5.65 to −0.89; Z = 2.70, *p* = 0.007), confirming the robustness of SEMA over the placebo effect on the VAT area (Figure 2, bottom).

One trial included two arms administering different weekly doses of SEMA 1.7 mg and 2.4 mg, both over 68 weeks [18]. As shown in Figure 2, the results from this study produced an SMD that was noticeably larger than the other four observations, where participants received SEMA doses of up to 1 mg.

##### 3.3.2.1 Sensitivity Analyses

The overall meta‐analysis yielded a significant result, indicating a meaningful VAT area reduction across observations. The sensitivity analysis demonstrated that the overall effect remained significant (*p* values between 0.009 and 0.03) for most exclusions, with effect sizes ranging from 2.12 to 2.63 and high heterogeneity (*I* ^2^ 98%–99%). However, excluding an observation derived from Kadowaki 2022’s study (SMD = −11.48), which was an outlier and related to the trial SEMA 2.4 mg, shifted the result to a nonsignificant *p* value of 0.06 and a reduced effect size of 1.87.

### 3.4 Meta‐Analyses of the Effect of SEMA on VAT Mass

#### 3.4.1 Pre‐Post SEMA

**FIGURE 4:**
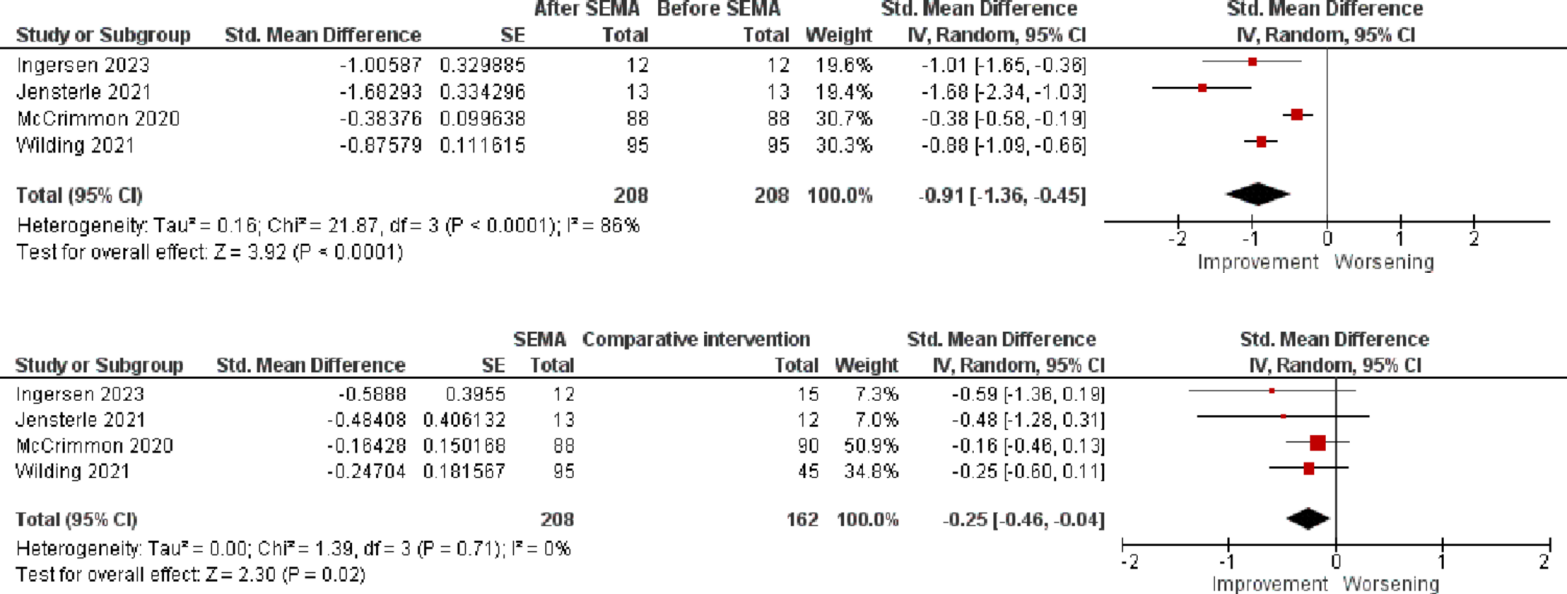
Forest plots of effects of SEMA on VAT volume.

There were four eligible studies for the pre‐post effect of SEMA on VAT mass. The ES was statistically significant and large (SMD = −0.91, 95% CI: −1.36 to −0.45; Z = 3.92, *p* < 0.0001; Figure 4, top).

##### 3.4.1.1 Sensitivity Analysis

In the sensitivity analysis, the effect remained significant across all iterations, suggesting that the main findings are robust and not significantly influenced by any single study.

#### 3.4.2 Pre‐Post SEMA vs. Comparative Intervention

Four studies comparing SEMA with other interventions (training, placebo, and Canagliflozin 300 mg) were eligible. As Figure 4 (bottom) shows, the effect size was overall significant (SMD = −0.25, 95% CI: −0.46 to −0.04; *p* = 0.02), but it was not significant for any of the single studies – SMD 95% CI crosses the zero line for all of them. This reflects the accumulated effect from all studies.

##### 3.4.2.1 Sensitivity Analysis

In the sensitivity analysis, excluding individual studies fluctuated the *p* value between 0.03 and 0.06. However, the effect size remained consistent, ranging from 1.86 to 2.17, and the heterogeneity (*I* ^2^ = 0%) did not change across the iterations. The marginal change in *p* value does not indicate that any study is particularly influential but rather confirms the accumulated effect from all studies.

### 3.5 Meta‐Analyses of the Effect of SEMA on VAT Volume

#### 3.5.1 Pre‐Post SEMA

**FIGURE 5:**
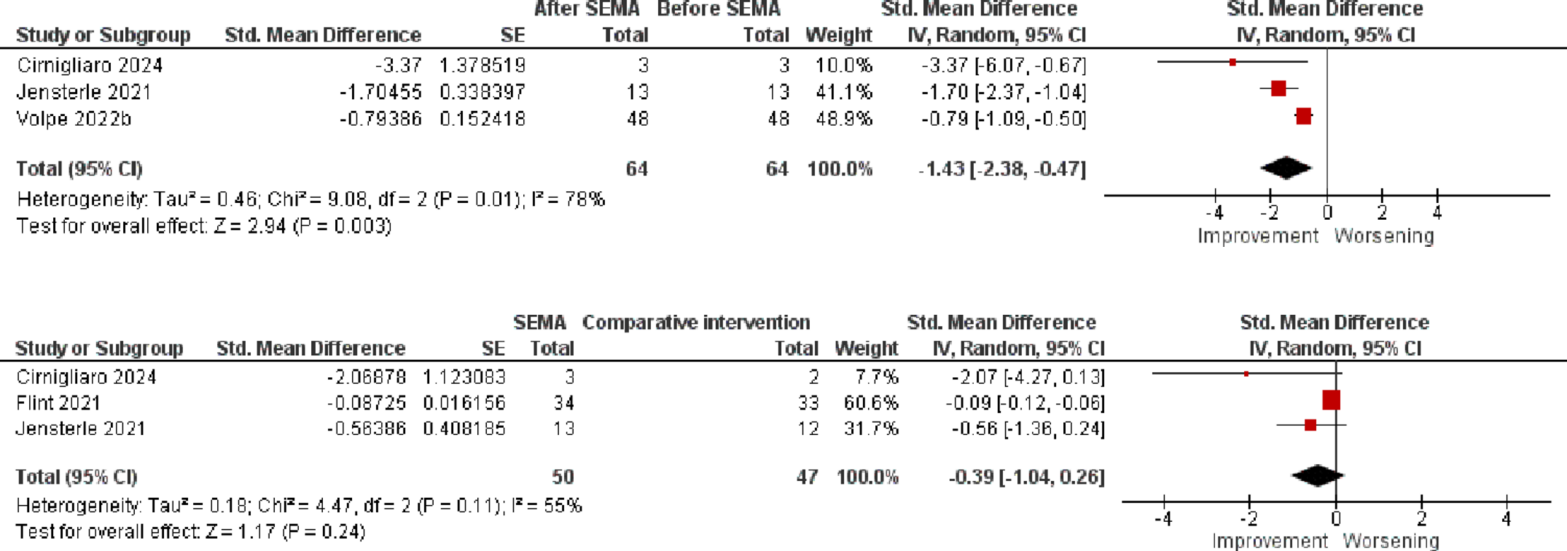
Forest plots of effects of SEMA on SAT area.

We found three eligible studies that assessed VAT volume before and after SEMA treatment. The effect size of −1.43 (95% CI: −2.38 to −0.47; Z = 2.94, *p* = 0.003) was in favor of the strong impact of SEMA on reducing VAT volume (Figure 5, top).

##### 3.5.1.1 Sensitivity Analysis

Excluding one of the studies shifted the significant effect (*p* = 0.003) to a nonsignificant result (*p* = 0.16). However, the effect size and confidence intervals of the excluded study were similar to those of the remaining studies. This suggests that the overall effect is robust and likely an accumulation of effects from all three studies.

#### 3.5.2 Pre‐Post SEMA vs. Comparative Intervention

The effect size calculated for the three studies was not significant (*p* = 0.24; Figure 5, bottom).

##### 3.5.2.1 Sensitivity Analysis

The overall effect remained insignificant when each observation was individually excluded (*p* values between 0.15 and 0.42).

### 3.6 Meta‐Analyses of the Effect of SEMA on SAT Area

#### 3.6.1 Pre‐Post SEMA

**FIGURE 6:**
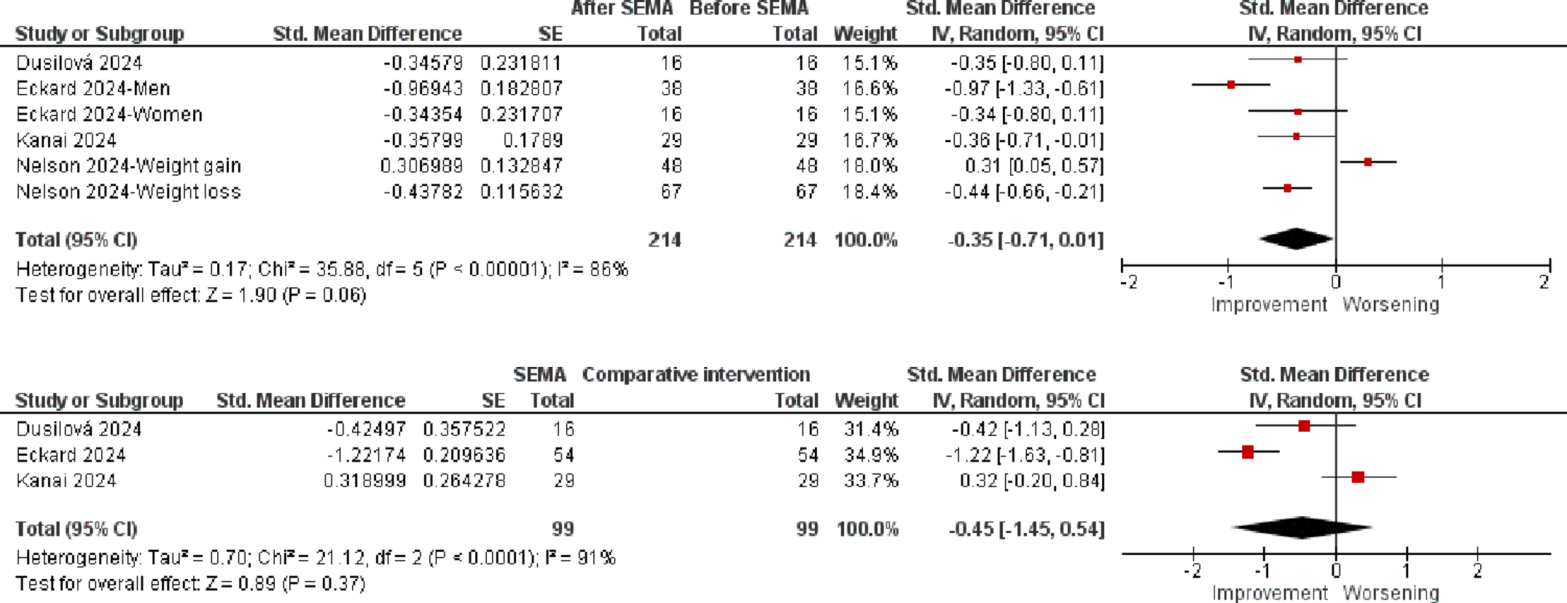
Meta‐regression plots of effects of SEMA on VAT area in single‐group comparisons.

There were six observations for the pre‐post effect of SEMA on the SAT area. The ES was not statistically significant (*p* = 0.06; Figure 6, top).

##### 3.6.1.1 Sensitivity Analysis

Excluding one specific observation derived from Nelson 2024’s study regarding patients who experienced weight gain with SEMA, the analysis became significant with an overall effect size of 4.26 (SMD = −0.50 [−0.73, −0.27], *p* < 0.0001) and heterogeneity was reduced (*I* ^2^ = 52%).

#### 3.6.2 Pre‐Post SEMA vs. Comparative Intervention

Three observations were included, but the ES was not significant (*p* = 0.37).

##### 3.6.2.1 Sensitivity Analysis

When an observation related to the Kanai 2024’s study of the impact of SEMA versus LSG was excluded, the analysis showed an overall significant effect size of 2.22 (SMD = −0.88 [−1.65, −0.10], *p* = 0.03) and a reduced heterogeneity (*I* ^2^ = 73%).

**FIGURE 7:**
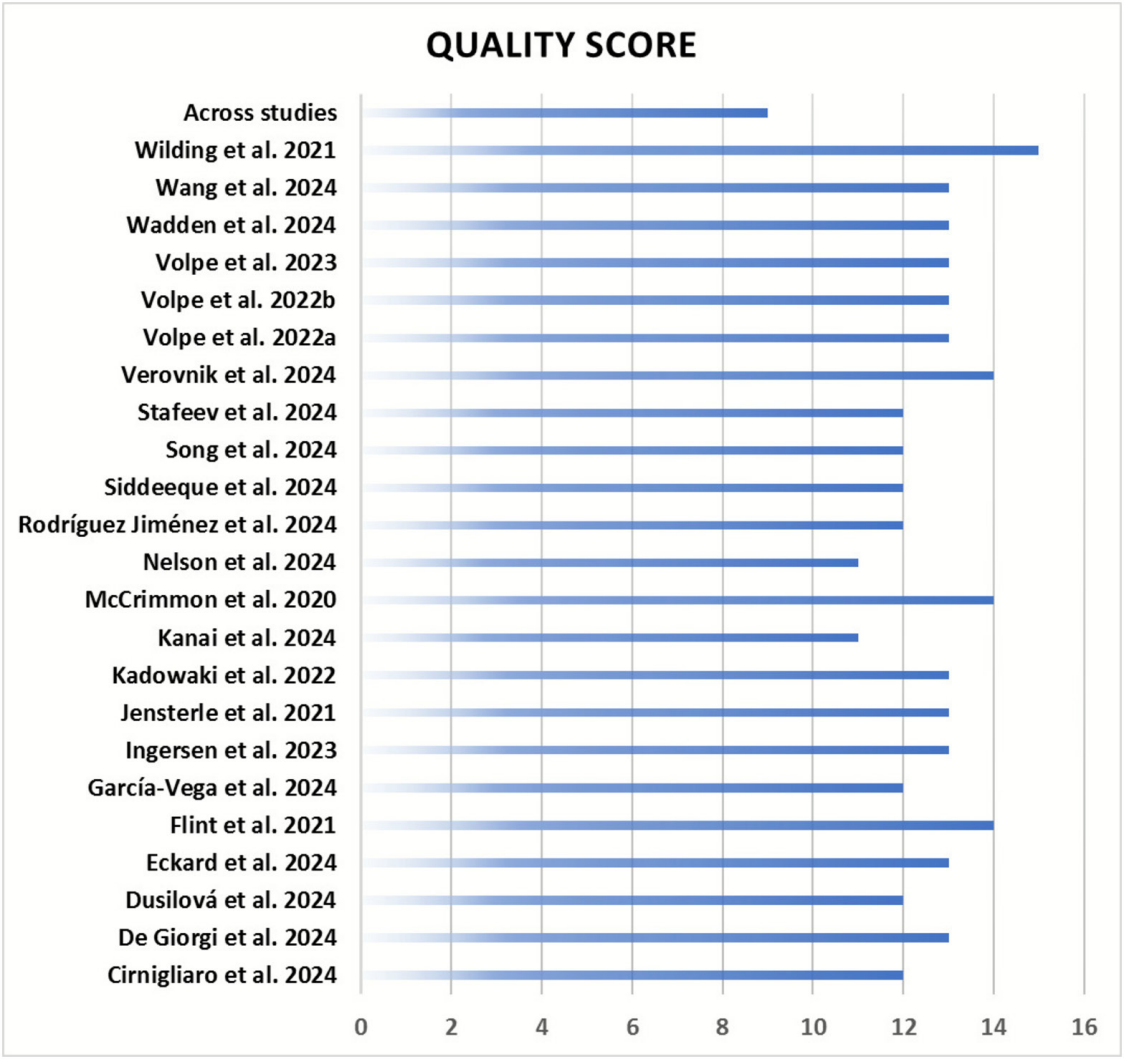
Summary of quality score of the included studies.

Figure 7 is a schema presenting the quality of the included studies.

## 4 Discussion

### 4.1 Summary and Interpretation of Findings

In this systematic review, we synthesized findings from 23 studies to comprehensively assess the effects of SEMA on SAT/VAT, brain function, and cognitive health. Our results indicate that SEMA significantly reduces VAT, as evidenced by reductions in area, mass, and volume. Additionally, sensitivity analyses suggested that SEMA might have led to a SAT area reduction. Moreover, a qualitative review of five studies revealed that SEMA appears to affect brain function over time and also to mitigate the risk of developing AD and other forms of dementia when compared to various alternative treatments. Meta‐analytic findings, built upon a context of highly heterogeneous original investigations, underscore the multifaceted benefits of SEMA in both metabolic and neurological health. The findings are explored in more detail in the following paragraphs.

Our findings revealed that SEMA could lead to improvements in VAT‐related indices, such as area, mass, and volume, with SMD between −1.43 and −0.91. The impact of SEMA on reducing VAT area and mass was also significant in two‐group comparisons, with SMDs of −3.27 and −0.25.

Through subgroup analyses in the meta‐analysis of single‐group comparisons of VAT area, we identified study design and measurement techniques as critical contributors to the observed variability. The differences were particularly pronounced in observational studies and RCTs, as well as in the methodologies used (CT and BIA).

Meta‐regression indicated a positive correlation between BMI and SMD, suggesting that individuals with higher BMI experience a less pronounced reduction in VAT. Conversely, longer duration was associated with a more significant reduction in VAT. The multivariate analysis confirmed the statistical significance of both BMI and duration as predictors of SMD, explaining 86.05% of the variability. The mixed‐effects model supported these findings by indicating no residual variability when considering BMI, duration, study design, and measurement technique, accounting for the whole variability.

Two meta‐analyses were conducted on the SAT area, initially yielding an insignificant effect size. After removing outliers, the overall SMD of −0.50 in single‐group comparisons and −0.88 in two‐group comparisons indicated that SEMA could be effective.

Only one study examined the impact of SEMA on brain function. SEMA led to a significant increase in RSFC in several brain regions over time. Four studies compared SEMA against various therapeutics, placebo, and no treatment concerning cognitive health (Supplemental Text 1). SEMA generally outperforms all the comparative drug classes (DPP‐4i, GLP‐1RAs, insulin, metformin, SGLT2i, TZD, SU) in reducing AD and other forms of dementia. The strongest effects are observed in women and individuals with obesity. The most effective comparisons for SEMA are with insulin, SUL, DPP‐4i, and TZD, where it consistently shows the largest reductions in AD risk, particularly in women. In SGLT2i and Empagliflozin comparisons, SEMA demonstrates stronger effects on dementia but no significant advantage over these treatments for cognitive deficit outcomes.

### 4.2 Appraisal of the Included Studies

As detailed in Supplemental Table 2, the quality assessment revealed that all studies were of high quality, with an average quality score of 12.7 (range: 11–15). The items that the studies scored low were mainly under the methodological domains, including sample size (justification), intervention (contamination and co‐intervention bias), and reporting (attrition bias).

### 4.3 Strengths and Limitations

This systematic review and meta‐analysis are the first to evaluate the influence of SEMA on outcomes related to both SAT and VAT, as well as its effects on brain function and cognition. In doing so, this review also integrates insights from the new definition of clinical obesity that goes beyond BMI to incorporate better measures of body fat distribution [46].

We conducted a comprehensive search and invested in data harmonization to gather sufficient information for evidence synthesis. Walking along this, we applied possible additional analyses to ensure the effects of heterogeneity (mediator, moderator, and sensitivity analyses) and potential publication bias (trim‐and‐fill method) are incorporated into adjusting or explaining the findings.

Our approach allowed for robust quantitative analysis of SAT/VAT‐related indices and a qualitative review of brain and cognitive function outcomes. Key findings indicate that BMI (Figure 3, top), duration (Figure 3, bottom), study design, and measurement techniques significantly moderate SEMA's influence on the VAT area. Validating these models is essential for informed decision‐making in disease management and prevention.

Although the included studies were of acceptable quality (Figure 7), the limited number of studies and small sample sizes restrict the reliability and generalizability of our findings. Previous meta‐analyses have highlighted factors such as gender, comorbid conditions, race/ethnicity, and BMI as potential sources of variability across studies. Here, inadequate data and the inclusion of fewer than 10 studies prevented the exploration of moderator analyses for heterogeneity and publication bias in most meta‐analyses. It is, therefore, essential to proceed carefully with the interpretation of findings from analyses where high heterogeneity was present (*I* ^2^ > 90%) and more specifically from those where explorative analyses were not executable due to the aforementioned reasons.

Missing information complicates meta‐analyses that analyze pre‐post effects, which require correlation coefficients. Only 27.8% of studies provided the necessary data for this calculation. Future updates should be considered as more original studies are published to enhance our understanding of these associations.

Exploring the mediation or moderation relationships between VAT and AD was not feasible due to the lack of overlapping data. Future studies examining the impact of SEMA on both outcomes could enable such analyses, enhancing our understanding of the potential connections between VAT and AD.

It is important to note that the studies included both individuals with T2D and those without, as the intervention is used in both contexts (weight loss and T2D management). This variability in study populations may influence the outcomes. Future studies should consider examining these populations separately to better understand the distinct effects in each group.

## 5 Conclusion

Our review systematically assessed the current literature on the topic and critically appraised highly heterogeneous original investigations. VAT was the most studied and consistently impacted outcome associated with SEMA treatment. Meta‐analyses demonstrated that SEMA may be able to promote the loss of both SAT and VAT; however, the effect seems to be particularly pronounced on VAT than SAT. Moreover, SEMA tends to outperform other drug classes in effectively reducing AD and other forms of dementia.

As we proceed with this meta‐analysis, it is crucial to consider whether the VAT decrease linked to the action of SEMA is associated with enhancements in cognitive function in the future.

## Conflicts of Interest

Dr. Cyrus Raji consults with Neurevolution LLC, Eli Lilly, Pacific Neuroscience Foundation, and Voxelwise LLC.

## Data Availability

Data for the meta‐analyses were derived from original publications and their associated files, including the main text, supplementary materials, and figures, or provided by their authors. They are compiled in a dataset specific to the project and are available upon reasonable request.
