# Supplementary material for: A Systematic Review and Meta-Analysis of Semaglutide Effects on Adipose Tissue and Emerging Effects on Brain and Cognition
Source: Obes Rev. Author manuscript; Available in PMC 2026 May 1. (PMC13131993; doi:10.1111/obr.70108)
Supplement: Supplementary file 1 [file NIHMS2154436-supplement-Supplemental_File_1.docx]

**A Systematic Review and Meta-analysis of Semaglutide Effects on Adipose Tissue and Emerging Effects on Brain and Cognition** 

Amene Saghazadeh^1^, Mahsa Dolatshahi^2^, Soheil Mohammadi^2^, Sara Hosseinzadeh Kassani^2^, Mahshid Naghashzadeh^2^, Joseph E. Ippolito^2,3^, Claude B. Sirlin^4^, Bettina Mittendorfer^5^, Matthew R. Brier^6^, Suzanne E. Schindler^6,7^, John C. Morris^6,7^, Danny Mou^8^, Hani Charles Soudah^9^, Tammie L. S. Benzinger^2,7,10^, Cyrus A. Raji^2,6,7^

1. *School of Medicine, Tehran University of Medical Sciences, Tehran, Iran* 

2. *Mallinckrodt Institute of Radiology, Washington University in St. Louis, St. Louis, Missouri, USA*

3. *Department of Biochemistry and Molecular Biophysics, Washington University School of Medicine, St. Louis, Missouri, USA* 

4. *Liver Imaging Group, Department of Radiology, University of California, San Diego, California, USA*

5. *Department of Medicine and Nutrition & Exercise Physiology, University of Missouri School of Medicine, Columbia, Missouri, USA*

6. *Department of Neurology, Washington University School of Medicine, St. Louis, Missouri, USA*

7. *Charles F. and Joanne Knight Alzheimer Disease Research Center (Knight ADRC), Washington University School of Medicine, St. Louis, Missouri, USA*

8. *Department of Surgery, Washington University School of Medicine, St. Louis, Missouri, USA*

9. *Department of Medicine, Washington University School of Medicine, St. Louis, Missouri, USA*

10. *Department of Neurosurgery, Washington University School of Medicine, St. Louis, Missouri, USA*

***Keywords:*** *Adiposity, BMI, Dementia, Diabetes, Incretin mimetic, Treatment duration, VAT area, Visceral obesity*

**Running title:** Semaglutide and visceral obesity

**Corresponding author:** Cyrus Raji, MD, PhD; E-mail: [craji@wustl.edu](mailto:craji@wustl.edu) 

**Acknowledgments:** The authors thank Dr. Eman A. Toraih for providing the data for their study.^1^ Dr. Cyrus Raji and his laboratory (Drs. Mahsa Dolatshahi, Soheil Mohammadi, and Mahshid Naghashzadeh) are supported in this work by funding from the National Institute of Aging (R01AG072637-01).

**Conflicts of interest statement:** Dr. Cyrus Raji consults with Neurevolution LLC. Eli Lilly, Pacific Neuroscience Foundation, and Voxelwise LLC.

# **Supplemental File 1 PRISMA Checklist**

| **Section and Topic** | **Item #** | **Checklist item** | **Location where item is reported** |
| --- | --- | --- | --- |
| **TITLE** |  |  |  |
| Title | 1 | Identify the report as a systematic review. | Page 1 |
| **ABSTRACT** |  |  |  |
| Abstract | 2 | See the PRISMA 2020 for Abstracts checklist. | Page 1 |
| **INTRODUCTION** |  |  |  |
| Rationale | 3 | Describe the rationale for the review in the context of existing knowledge. | Page 2 |
| Objectives | 4 | Provide an explicit statement of the objective(s) or question(s) the review addresses. | Page 2 |
| **METHODS** |  |  |  |
| Eligibility criteria | 5 | Specify the inclusion and exclusion criteria for the review and how studies were grouped for the syntheses. | Pages 2 |
| Information sources | 6 | Specify all databases, registers, websites, organisations, reference lists and other sources searched or consulted to identify studies. Specify the date when each source was last searched or consulted. | Page 2 |
| Search strategy | 7 | Present the full search strategies for all databases, registers and websites, including any filters and limits used. | Supplemental File 2 |
| Selection process | 8 | Specify the methods used to decide whether a study met the inclusion criteria of the review, including how many reviewers screened each record and each report retrieved, whether they worked independently, and if applicable, details of automation tools used in the process. | Page 2 |
| Data collection process | 9 | Specify the methods used to collect data from reports, including how many reviewers collected data from each report, whether they worked independently, any processes for obtaining or confirming data from study investigators, and if applicable, details of automation tools used in the process. | Page 3 |
| Data items | 10a | List and define all outcomes for which data were sought. Specify whether all results that were compatible with each outcome domain in each study were sought (e.g. for all measures, time points, analyses), and if not, the methods used to decide which results to collect. | Pages 2 and 3 |
|  | 10b | List and define all other variables for which data were sought (e.g. participant and intervention characteristics, funding sources). Describe any assumptions made about any missing or unclear information. | Pages 2 and 3 |
| Study risk of bias assessment | 11 | Specify the methods used to assess risk of bias in the included studies, including details of the tool(s) used, how many reviewers assessed each study and whether they worked independently, and if applicable, details of automation tools used in the process. | Page 3 |
| Effect measures | 12 | Specify for each outcome the effect measure(s) (e.g. risk ratio, mean difference) used in the synthesis or presentation of results. | Page 3 |
| Synthesis methods | 13a | Describe the processes used to decide which studies were eligible for each synthesis (e.g. tabulating the study intervention characteristics and comparing against the planned groups for each synthesis (item #5)). | Page 3 |
|  | 13b | Describe any methods required to prepare the data for presentation or synthesis, such as handling of missing summary statistics, or data conversions. | Page 3 |
|  | 13c | Describe any methods used to tabulate or visually display results of individual studies and syntheses. | Page 3 |
|  | 13d | Describe any methods used to synthesize results and provide a rationale for the choice(s). If meta-analysis was performed, describe the model(s), method(s) to identify the presence and extent of statistical heterogeneity, and software package(s) used. | Page 3 |
|  | 13e | Describe any methods used to explore possible causes of heterogeneity among study results (e.g. subgroup analysis, meta-regression). | Page 3 |
|  | 13f | Describe any sensitivity analyses conducted to assess robustness of the synthesized results. | Page 3 |
| Reporting bias assessment | 14 | Describe any methods used to assess risk of bias due to missing results in a synthesis (arising from reporting biases). | Page 3 |
| Certainty assessment | 15 | Describe any methods used to assess certainty (or confidence) in the body of evidence for an outcome. | Page 3 |
| **RESULTS** |  |  |  |
| Study selection | 16a | Describe the results of the search and selection process, from the number of records identified in the search to the number of studies included in the review, ideally using a flow diagram. | Page 4 |
|  | 16b | Cite studies that might appear to meet the inclusion criteria, but which were excluded, and explain why they were excluded. | Page 4 |
| Study characteristics | 17 | Cite each included study and present its characteristics. | Pages 4-9, Table 1, Supplemental Table 1 |
| Risk of bias in studies | 18 | Present assessments of risk of bias for each included study. | Page 16, Supplemental Table 2 |
| Results of individual studies | 19 | For all outcomes, present, for each study: (a) summary statistics for each group (where appropriate) and (b) an effect estimate and its precision (e.g. confidence/credible interval), ideally using structured tables or plots. | Pages 4-9, Table 1, Supplemental Table 1 |
| Results of syntheses, | 20a | For each synthesis, briefly summarise the characteristics and risk of bias among contributing studies. | Pages 10-16, Supplemental Text 1 |
|  | 20b | Present results of all statistical syntheses conducted. If meta-analysis was done, present for each the summary estimate and its precision (e.g. confidence/credible interval) and measures of statistical heterogeneity. If comparing groups, describe the direction of the effect. | Pages 10-16, Supplemental Text 1 |
|  | 20c | Present results of all investigations of possible causes of heterogeneity among study results. | Pages 10-16, Supplemental Text 1 |
|  | 20d | Present results of all sensitivity analyses conducted to assess the robustness of the synthesized results. | Pages 10-16, Supplemental Text 1 |
| Reporting biases | 21 | Present assessments of risk of bias due to missing results (arising from reporting biases) for each synthesis assessed. | Pages 10-16 |
| Certainty of evidence | 22 | Present assessments of certainty (or confidence) in the body of evidence for each outcome assessed. | Pages 10-16 |
| **DISCUSSION** |  |  |  |
| Discussion | 23a | Provide a general interpretation of the results in the context of other evidence. | Pages 16-18 |
|  | 23b | Discuss any limitations of the evidence included in the review. | Pages 16-18 |
|  | 23c | Discuss any limitations of the review processes used. | Pages 16-18 |
|  | 23d | Discuss implications of the results for practice, policy, and future research. | Pages 16-18 |
| **OTHER INFORMATION** |  |  |  |
| Registration and protocol | 24a | Provide registration information for the review, including register name and registration number, or state that the review was not registered. | Not registered |
|  | 24b | Indicate where the review protocol can be accessed, or state that a protocol was not prepared. | Available on request |
|  | 24c | Describe and explain any amendments to information provided at registration or in the protocol. | Not applicable |
| Support | 25 | Describe sources of financial or non-financial support for the review, and the role of the funders or sponsors in the review. | Page 18 |
| Competing interests | 26 | Declare any competing interests of review authors. | Page 18 |
| Availability of data, code and other materials | 27 | Report which of the following are publicly available and where they can be found: template data collection forms; data extracted from included studies; data used for all analyses; analytic code; any other materials used in the review. | Page 18 |

# **Supplemental File 2 Search strategy** 

- **Scopus**

TITLE-ABS ( Semaglutide  OR  Ozempic  OR  Rybelsus  OR  Wegovy  OR  OG-217SC  OR  NN-9924  OR  NN9924  OR  OG217SC )  AND  TITLE-ABS ( Brain  OR  head  OR  cranial  OR  cerebral  OR  alzheim*  OR  dement*  OR  cognit*  OR  neuro*  OR  psych*  OR  neuropsych*  OR  Memory  OR  imaging  OR  fMRI  OR  adipos*  OR  fat  OR  “magnetic resonance”  OR  tomography  OR  dexa  OR  absorptiometry  OR  decisio*  OR  execut*  OR  functio*  OR  perfor*  OR  processing  OR  attenti*  OR  learning  OR  impuls*  OR  rewar* )

Date of last search: November 09, 2024

Filters applied: English

Number of results: 763

- **PubMed**

(Semaglutide[Title/Abstract] OR Ozempic[Title/Abstract] OR Rybelsus[Title/Abstract] OR Wegovy[Title/Abstract] OR OG-217SC[Title/Abstract] OR NN-9924[Title/Abstract] OR NN9924[Title/Abstract] OR OG217SC[Title/Abstract]) AND (Brain[Title/Abstract] OR head[Title/Abstract] OR cranial[Title/Abstract] OR cerebral[Title/Abstract] OR Alzheim*[Title/Abstract] OR dement*[Title/Abstract] OR Cognit*[Title/Abstract] OR neuro*[Title/Abstract] OR psych*[Title/Abstract] OR neuropsych*[Title/Abstract] OR Memory[Title/Abstract] OR imaging[Title/Abstract] OR fMRI[Title/Abstract] OR adipos*[Title/Abstract] OR fat[Title/Abstract] OR “magnetic resonance”[Title/Abstract] OR tomography[Title/Abstract] OR dexa[Title/Abstract] OR absorptiometry[Title/Abstract] OR decisio*[Title/Abstract] OR execut*[Title/Abstract] OR functio*[Title/Abstract] OR perfor*[Title/Abstract] OR processing[Title/Abstract] OR attenti*[Title/Abstract] OR learning[Title/Abstract] OR impuls*[Title/Abstract] OR rewar*[Title/Abstract])

Date of last search: November 09, 2024

Filters applied: English

Number of results: 806  

- **Web of Science** 

(TS=(Semaglutide OR Ozempic OR Rybelsus OR Wegovy OR OG-217SC OR NN-9924 OR NN9924 OR OG217SC )) AND TS=(Brain OR head OR cranial OR cerebral OR Alzheim* OR dement* OR Cognit* OR neuro* OR psych* OR neuropsych* OR Memory OR imaging OR fMRI OR adipos* OR fat OR “magnetic resonance” OR tomography OR dexa OR absorptiometry OR decisio* OR execut* OR functio* OR perfor* OR processing OR attenti* OR learning OR impuls* OR rewar*)

Date of last search: March 27, 2023

Number of results: 427

- **Embase**

(semaglutide:ab,ti OR ozempic:ab,ti OR rybelsus:ab,ti OR wegovy:ab,ti OR og-217sc:ab,ti OR nn-9924:ab,ti OR nn9924:ab,ti OR og217sc:ab,ti) AND (brain:ab,ti OR head:ab,ti OR cranial:ab,ti OR cerebral:ab,ti OR alzheim*:ab,ti OR dement*:ab,ti OR cognit*:ab,ti OR neuro*:ab,ti OR psych*:ab,ti OR neuropsych*:ab,ti OR memory:ab,ti OR imaging:ab,ti OR fmri:ab,ti OR adipos*:ab,ti OR fat:ab,ti OR ‘magnetic resonance’:ab,ti OR tomography:ab,ti OR dexa:ab,ti OR absorptiometry:ab,ti OR decisio*:ab,ti OR execut*:ab,ti OR functio*:ab,ti OR perfor*:ab,ti OR processing:ab,ti OR attenti*:ab,ti OR learning:ab,ti OR impuls*:ab,ti OR rewar*:ab,ti) 

Date of last search: March 27, 2023

Number of results: 658 

# **Supplemental Table 1 Summary of observations derived from studies evaluating the effect of SEMA in the risk of developing Alzheimer’s disease and other forms of dementia** 

| **Study** | **Comparison** | **Sample size** | **Outcome** | **Log HR** | **SE Log HR** | **95% CI – LL** | **95% CI – UL** | **Significance of effect** | **Magnitude of effect** |
| --- | --- | --- | --- | --- | --- | --- | --- | --- | --- |
| De Giorgi et al. 2024 | SEMA vs. Sitagliptin | 46772 | Dementia | -0.65 | 0.14 | -0.92 | -0.39 | Yes | Strong |
| De Giorgi et al. 2024 | SEMA vs. Glipizide | 38412 | Dementia | -0.46 | 0.16 | -0.77 | -0.15 | Yes | Moderate |
| De Giorgi et al. 2024 | SEMA vs. Sitagliptin | 46772 | Cognitive deficit | -0.33 | 0.06 | -0.44 | -0.22 | Yes | Moderate |
| De Giorgi et al. 2024 | SEMA vs. Glipizide | 38412 | Cognitive deficit | -0.33 | 0.06 | -0.45 | -0.2 | Yes | Moderate |
| De Giorgi et al. 2024 | SEMA vs. Empagliflozin | 45168 | Dementia | -0.09 | 0.14 | -0.38 | 0.19 | No | Not significant |
| De Giorgi et al. 2024 | SEMA vs. Empagliflozin | 45168 | Cognitive deficit | -0.04 | 0.06 | -0.15 | 0.07 | No | Not significant |
| Siddeeque et al. 2024 | Semaglutide vs none | 114086 | VD | -1.7 | 0.31 | -2.32 | -1.09 | Yes | Strong |
| Siddeeque et al. 2024 | Semaglutide vs none | 114086 | AD | -0.92 | 0.23 | -1.37 | -0.48 | Yes | Strong |
| Siddeeque et al. 2024 | Semaglutide vs none | 114086 | LBD | -0.89 | 0.21 | -1.3 | -0.48 | Yes | Strong |
| Wadden et al. 2024 | SEMA vs. placebo | 3681 | Cognitive and attention disorders and disturbances | -0.19 | 0.76 | -1.68 | 1.31 | No | Not significant |
| Wang et al. 2024 | SEMA vs. Insulin-Women | 17762 | AD | -1.51 | 0.32 | -2.14 | -0.89 | Yes | Strong |
| Wang et al. 2024 | SEMA vs. Insulin-With obesity | 19066 | AD | -1.24 | 0.34 | -1.91 | -0.56 | Yes | Strong |
| Wang et al. 2024 | SEMA vs. TZD-Women | 9988 | AD | -1.2 | 0.35 | -1.9 | -0.51 | Yes | Strong |
| Wang et al. 2024 | SEMA vs. SU-Total | 33006 | AD | -1.17 | 0.22 | -1.61 | -0.73 | Yes | Strong |
| Wang et al. 2024 | SEMA vs. SU-With obesity | 17734 | AD | -1.17 | 0.36 | -1.87 | -0.47 | Yes | Strong |
| Wang et al. 2024 | SEMA vs. TZD-With obesity | 8512 | AD | -1.17 | 0.44 | -2.04 | -0.3 | Yes | Strong |
| Wang et al. 2024 | SEMA vs. Insulin-Without obesity | 15088 | AD | -1.14 | 0.29 | -1.72 | -0.56 | Yes | Strong |
| Wang et al. 2024 | SEMA vs. Insulin-Total | 34174 | AD | -1.11 | 0.23 | -1.55 | -0.67 | Yes | Strong |
| Wang et al. 2024 | SEMA vs. Metformin-Women | 17750 | AD | -1.11 | 0.33 | -1.76 | -0.45 | Yes | Strong |
| Wang et al. 2024 | SEMA vs. SU-Women | 16912 | AD | -1.11 | 0.33 | -1.76 | -0.45 | Yes | Strong |
| Wang et al. 2024 | SEMA vs. TZD-Without obesity | 12844 | AD | -1.11 | 0.3 | -1.7 | -0.52 | Yes | Strong |
| Wang et al. 2024 | SEMA vs. SU-Without obesity | 15064 | AD | -1.05 | 0.29 | -1.62 | -0.48 | Yes | Strong |
| Wang et al. 2024 | SEMA vs. DPP-4i-Women | 16142 | AD | -1.02 | 0.35 | -1.7 | -0.34 | Yes | Strong |
| Wang et al. 2024 | SEMA vs. Metformin-Total | 34160 | AD | -0.97 | 0.23 | -1.42 | -0.52 | Yes | Strong |
| Wang et al. 2024 | SEMA vs. DPP-4i-Men | 13258 | AD | -0.94 | 0.33 | -1.6 | -0.29 | Yes | Strong |
| Wang et al. 2024 | SEMA vs. DPP-4i-Total | 31756 | AD | -0.92 | 0.23 | -1.36 | -0.47 | Yes | Strong |
| Wang et al. 2024 | SEMA vs. SU-Men | 13700 | AD | -0.92 | 0.33 | -1.57 | -0.27 | Yes | Strong |
| Wang et al. 2024 | SEMA vs. Metformin-Without obesity | 15096 | AD | -0.89 | 0.29 | -1.47 | -0.31 | Yes | Strong |
| Wang et al. 2024 | SEMA vs. TZD-Total | 20974 | AD | -0.84 | 0.25 | -1.34 | -0.35 | Yes | Strong |
| Wang et al. 2024 | SEMA vs. Metformin-Men | 13782 | AD | -0.8 | 0.34 | -1.46 | -0.14 | Yes | Strong |
| Wang et al. 2024 | SEMA vs. Metformin-With obesity | 19060 | AD | -0.8 | 0.36 | -1.51 | -0.08 | Yes | Strong |
| Wang et al. 2024 | SEMA vs. SGLT2i-Women | 14640 | AD | -0.78 | 0.36 | -1.49 | -0.07 | Yes | Strong |
| Wang et al. 2024 | SEMA vs. DPP-4i-Without obesity | 15014 | AD | -0.76 | 0.31 | -1.35 | -0.16 | Yes | Strong |
| Wang et al. 2024 | SEMA vs. SGLT2i-With obesity | 14938 | AD | -0.73 | 0.39 | -1.51 | 0.04 | No | Not significant |
| Wang et al. 2024 | SEMA vs. Insulin-Men | 13790 | AD | -0.73 | 0.35 | -1.41 | -0.06 | Yes | Strong |
| Wang et al. 2024 | SEMA vs. DPP-4i-With obesity | 16448 | AD | -0.69 | 0.38 | -1.43 | 0.04 | No | Not significant |
| Wang et al. 2024 | SEMA vs. Other GLP-1RAs-Women | 17728 | AD | -0.63 | 0.36 | -1.34 | 0.07 | No | Not significant |
| Wang et al. 2024 | SEMA vs. Other GLP-1RAs-Without obesity | 15078 | AD | -0.62 | 0.31 | -1.23 | 0 | Yes | Strong |
| Wang et al. 2024 | SEMA vs. SGLT2i-Without obesity | 15078 | AD | -0.62 | 0.31 | -1.23 | 0 | Yes | Strong |
| Wang et al. 2024 | SEMA vs. Other GLP-1RAs-With obesity | 18896 | AD | -0.53 | 0.38 | -1.28 | 0.22 | No | Not significant |
| Wang et al. 2024 | SEMA vs. Other GLP-1RAs-Total | 34418 | AD | -0.53 | 0.24 | -1 | -0.06 | Yes | Strong |
| Wang et al. 2024 | SEMA vs. SGLT2i-Total | 30576 | AD | -0.51 | 0.25 | -1 | -0.02 | Yes | Strong |
| Wang et al. 2024 | SEMA vs. TZD-Men | 10700 | AD | -0.34 | 0.37 | -1.07 | 0.39 | No | Not significant |
| Wang et al. 2024 | SEMA vs. Other GLP-1RAs-Men | 13762 | AD | 0.13 | 0.41 | -0.67 | 0.94 | No | Not significant |
| Wang et al. 2024 | SEMA vs. SGLT2i-Men | 13762 | AD | 0.13 | 0.41 | -0.67 | 0.94 | No | Not significant |

# **Supplemental Table 2 Quality of the studies included in the systematic review**

| **Domain** | **Item** | **Cirnigliaro et al. 2024** | **De Giorgi et al. 2024** | **Dusilová et al. 2024** | **Eckard et al. 2024** | **Flint et al. 2021** | **García-Vega et al. 2024** | **Ingersen et al. 2023** | **Jensterle et al. 2021** | **Kadowaki et al. 2022** | **Kanai et al. 2024** | **McCrimmon et al. 2020** | **Nelson et al. 2024** | **Rodríguez Jiménez et al. 2024** | **Siddeeque et al. 2024** | **Song et al. 2024** | **Stafeev et al. 2024** | **Verovnik et al. 2024** | **Volpe et al. 2022a** | **Volpe et al. 2022b** | **Volpe et al. 2023** | **Wadden et al. 2024** | **Wang et al. 2024** | **Wilding et al. 2021** | **Across studies** |
| --- | --- | --- | --- | --- | --- | --- | --- | --- | --- | --- | --- | --- | --- | --- | --- | --- | --- | --- | --- | --- | --- | --- | --- | --- | --- |
| **STUDY PURPOSE** | **Was the purpose stated clearly?** | Yes | Yes | Yes | Yes | Yes | Yes | Yes | Yes | Yes | Yes | Yes | Yes | Yes | Yes | Yes | Yes | Yes | Yes | Yes | Yes | Yes | Yes | Yes | Yes |
| **LITERATURE** | **Was relevant background literature reviewed?** | Yes | Yes | Yes | Yes | Yes | Yes | Yes | Yes | Yes | Yes | Yes | Yes | Yes | Yes | Yes | Yes | Yes | Yes | Yes | Yes | Yes | Yes | Yes | Yes |
| **DESIGN** | **Randomized (RCT), cohort, single case design, before and after, case-control, cross-sectional, case study** | Case series | Retrospective | Cross-over | RCT | RCT | Observational | RCT | RCT | RCT | Retrospective | RCT | Retrospective | Quasi-experimental | Retrospective | Retrospective | Observational | RCT | Prospective, single-arm real-life | Prospective, single-arm real-life | Prospective, single-arm real-life | Post-hoc analysis | Emulation target trials | RCT | Can’t tell |
| **SAMPLE** | **Was the sample described in detail?** | Yes | Yes | Yes | Yes | No | Yes | Yes | No | Yes | Yes | No | No | Yes | Yes | Yes | Yes | Yes | No | Yes | Yes | Yes | Yes | Yes | Can’t tell |
|  | **Was sample size justified?** | No | Yes | No | Yes | Yes | No | Yes | No | Yes | No | Yes | Yes | Yes | Yes | No | No | Yes | Yes | Yes | Yes | Yes | Yes | Yes | Can’t tell |
| **OUTCOMES** | **Were the outcome measures reliable?** | Yes | Yes | Yes | Yes | Yes | Yes | Yes | Yes | Yes | Yes | Yes | Yes | Yes | Yes | Yes | Yes | Yes | Yes | Yes | Yes | Yes | Yes | Yes | Yes |
|  | **Were the outcome measures valid?** | Yes | Yes | Yes | Yes | Yes | Yes | Yes | Yes | Yes | Yes | Yes | Yes | Yes | Yes | Yes | Yes | Yes | Yes | Yes | Yes | Yes | Yes | Yes | Yes |
| **INTERVENTION** | **Intervention was described in detail?** | Yes | Yes | Yes | Yes | Yes | Yes | Yes | Yes | Yes | Yes | Yes | No | Yes | Yes | Yes | Yes | Yes | Yes | Yes | Yes | Yes | Yes | Yes | Yes |
|  | **Contamination was avoided?** | Not addressed | Yes | No | Yes | Yes | No | No | Yes | Yes | N/A | Yes | N/A | N/A | N/A | N/A | N/A | Yes | N/A | N/A | N/A | N/A | N/A | Yes | Can’t tell |
|  | **Co-intervention was avoided?** | Yes | No | No | Not addressed | Yes | Yes | No | Yes | Not addressed | Not addressed | Yes | Not addressed | No | N/A | Yes | No | No | Yes | Yes | Yes | No | Yes | Yes | Can’t tell |
| **RESULTS** | **Results were reported in terms of statistical significance?** | Yes | Yes | Yes | Yes | Yes | Yes | Yes | Yes | Yes | Yes | Yes | Yes | Yes | Yes | Yes | Yes | Yes | Yes | Yes | Yes | Yes | Yes | Yes | Yes |
|  | **Were the analysis method(s) appropriate?** | Yes | Yes | Yes | Yes | Yes | Yes | Yes | Yes | Yes | Yes | Yes | Yes | Yes | Yes | Yes | Yes | Yes | Yes | Yes | Yes | Yes | Yes | Yes | Yes |
|  | **Clinical importance was reported?** | Yes | Yes | Yes | Yes | Yes | Yes | Yes | Yes | Yes | Yes | Yes | Yes | Yes | Yes | Yes | Yes | Yes | Yes | Yes | Yes | Yes | Yes | Yes | Yes |
|  | **Drop-outs were reported?** | No | No | Yes | Yes | Yes | No | Yes | Yes | Yes | No | Yes | Yes | No | No | Yes | Yes | Yes | Yes | Yes | Yes | Yes | Yes | Yes | Can’t tell |
| **CONCLUSIONS AND IMPLICATIONS** | **Conclusions were appropriate given study methods and results** | Yes | Yes | Yes | Yes | Yes | Yes | Yes | Yes | Yes | Yes | Yes | Yes | Yes | Yes | Yes | Yes | Yes | Yes | Yes | Yes | Yes | Yes | Yes | Yes |
| **Quality score** |  | 12 | 13 | 12 | 13 | 14 | 12 | 13 | 13 | 13 | 11 | 14 | 11 | 12 | 12 | 12 | 12 | 14 | 13 | 13 | 13 | 13 | 13 | 15 | 9 |
| **Risk of bias** |  | Low | Low | Low | Low | Low | Low | Low | Low | Low | Low | Low | Low | Low | Low | Low | Low | Low | Low | Low | Low | Low | Low | Low |  |

# **Supplemental Figure 1 Funnel plot for publication bias in the meta-analysis of pre-post SEMA effects on the VAT area**

# **Supplemental Text 1: Supplemental Results**

## 1.1. Qualitative synthesis of the effect of SEMA on brain function

### 1.1.1.  **Pre-post SEMA**

An examination of the variations in RSFC across two visits (baseline and week 16) revealed significant differences.^1^ The SEMA group showed an increase in RSFC following the intervention, involving the Left Insula – Left Amygdala, Left Insula – Right Amygdala, Right Insula – Left Amygdala, Right Insula – Right Amygdala, and Left Parahippocampus – Left Amygdala. There were only two ROIs people in this group who showed a decrease in RSFC, including Left MTG – Right MTG and Right Precuneus – Left Insula. 

The placebo group experienced a decline in RSFC across some ROIs like Left MTG – Left Precuneus, Left MTG – Right Precuneus, Left Precuneus – Left Cingulate gyrus, Left Precuneus – Right Cingulate gyrus, Right Precuneus – Left Cingulate gyrus, Right Precuneus – Right Cingulate gyrus. Regions of interest with increased RSFC in the placebo group were Right Precuneus – Right Amygdala and Left Insula – Left Amygdala. 

The authors collectively concluded that SEMA generally increased RSFC over time, while the placebo tended to decrease it in the tested regions of interest.

### 1.1.2. **Pre-post SEMA vs. comparative intervention**

Comparisons between baseline measurements and those taken at week 16 showed no changes in RSFC with SEMA when compared to the placebo.^1^

## 1.2. Qualitative synthesis of the effect of SEMA on cognitive function

In four studies included in this systematic review, the risk of developing the outcomes of interest was compared between participants treated with SEMA and those in other intervention arms (Supplemental Table 1).^2-5^

### 1.2.1. **SEMA vs. DPP-4i**

Log HRs between -0.69 and -1.02 suggest that SEMA consistently reduces AD risk in comparison to DPP-4i across all subgroups (with obesity, without obesity, women, and men), with a stronger effect observed in women (log HR = -1.02). The effect is less pronounced in individuals with obesity (log HR = -0.69) but still significant. When particularly compared to Sitagliptin, SEMA shows a stronger reduction in dementia than in cognitive deficits (log HR of -0.65 vs. -0.33).

### 1.2.2. **SEMA vs. other GLP-1RAs**

SEMA shows a moderate reduction in AD risk compared to other GLP-1RAs (log HR between -0.63 to -0.53), with the most pronounced effect in women and individuals without obesity (log HR = -0.63 to -0.62). Men, however, show little to no difference (log HR = 0.13).

### 1.2.3. **SEMA vs. Insulin** 

With log HRs between -1.51 to -0.73, SEMA shows a strong reduction in AD risk compared to Insulin, particularly in women and individuals with obesity (log HR = -1.51 to -1.14). Men still see a significant benefit, but the effect is weaker (log HR = -0.73).

### 1.2.4. **SEMA vs. Metformin**

SEMA shows a moderate reduction in AD risk compared to Metformin (log HRs between -1.11 and -0.80), with the strongest effect seen in women.

### 1.2.5. **SEMA vs. none or placebo**

Log HRs ranging from -1.09 to -0.48 indicate a significant and strong effect of SEMA in reducing the risk of AD and other forms of dementia compared to no intervention. However, in another study, SEMA did not demonstrate a significant effect in reducing the risk of developing cognitive and attention disorders when compared to a placebo.

### 1.2.6. **SEMA vs. SGLT2i**

SEMA shows a moderate reduction in AD risk compared to SGLT2i (log HR between -0.77 to -0.51), especially for women and those with obesity. The effect is negligible for men (log HR of 0.13). Specifically, SEMA does not show a significant advantage over Empagliflozin for reducing cognitive deficits or dementia (log HR of -0.09 to -0.04).

### 1.2.7. **SEMA vs. Sulfonylureas**

SEMA shows a strong reduction in AD risk compared to SU (log HR between -1.17 and -0.92), particularly in those with obesity, with the effect being moderate for both men and women. Particularly, SEMA is more effective than Glipizide in reducing cognitive deficits and dementia, with a stronger effect on dementia (log HR of -0.46 vs. -0.33).

### 1.2.8. **SEMA vs. Thiazolidinediones**

SEMA consistently shows a strong reduction in AD risk compared to TZD (log HR between -1.20 and -0.84), especially in women (log HR = -1.20) and individuals with obesity (log HR = -1.17).

1.	Verovnik B, Vovk A. Semaglutide, suicidal ideation and behaviour: A resting state functional magnetic resonance imaging perspective. Letter. *Diabetes Obes Metab*. 2024;26(2):782-784. doi:10.1111/dom.15363

2.	De Giorgi R, Koychev I, Adler AI, et al. 12-month neurological and psychiatric outcomes of semaglutide use for type 2 diabetes: a propensity-score matched cohort study. Article. *EClinicalMedicine*. 2024;74102726. doi:10.1016/j.eclinm.2024.102726

3.	Siddeeque N, Hussein MH, Abdelmaksoud A, et al. Neuroprotective effects of GLP-1 receptor agonists in neurodegenerative Disorders: A Large-Scale Propensity-Matched cohort study. Article. *Int Immunopharmacol*. 2024;143113537. doi:10.1016/j.intimp.2024.113537

4.	Wadden TA, Brown GK, Egebjerg C, et al. Psychiatric Safety of Semaglutide for Weight Management in People Without Known Major Psychopathology Post Hoc Analysis of the STEP 1, 2, 3, and 5 Trials. Article. *JAMA Internal Medicine*. 2024;doi:10.1001/jamainternmed.2024.4346

5.	Wang W, Wang Q, Qi X, et al. Associations of semaglutide with first-time diagnosis of Alzheimer's disease in patients with type 2 diabetes: Target trial emulation using nationwide real-world data in the US. Article. *Alzheimer's Dementia*. 2024;doi:10.1002/alz.14313
